# Gossip promotes cooperation only when it is pro-socially motivated

**DOI:** 10.1038/s41598-022-08670-7

**Published:** 2022-03-21

**Authors:** Martina Testori, Charlotte K. Hemelrijk, Bianca Beersma

**Affiliations:** 1 Department of Organizational Sciences, Vrije Universiteit Amsterdam, De Boelelaan 1105, Amsterdam, 1081HV The Netherlands; 2 GELIEF, University of Groningen, Nijenborgh 7, Groningen, 9747AG The Netherlands

**Keywords:** Psychology, Human behaviour

## Abstract

Humans are often shown to cooperate with one another. Most of the mechanisms that foster cooperation among humans rely on reputation, which itself relies on the acquisition of information about other people’s behaviors. Gossip has been proposed as a cheap yet efficient tool to acquire information, and it has largely been proved to be an effective means to foster and maintain cooperation. However, empirical studies supporting this claim have ignored two aspects: (1) they often compared gossip to treatments in which no reputation was available, impeding a direct assessment of whether it is gossip that promotes cooperation or rather the introduction of a reputation system; and (2) they focused on pro-social gossip (e.g., gossip aimed at helping the receiver), neglecting the impact of other types of gossip. We show here that, in contrast with the widespread notion that gossip promotes cooperation, gossip mostly depletes cooperation compared to first-hand information. If lying is fruitful for individuals or if a group’s behavior is largely uncooperative, gossip leads to negative reputational information and decreased cooperation.

## Introduction

Cooperation with genetically unrelated others is widely observed among humans^1^. The main mechanisms proposed to foster cooperation among non-kin are direct reciprocity^2^ (i.e., “I help you, and you will help me”), indirect reciprocity^3,4^ (i.e., “I help you now, and someone else will help me in the future”), and competitive altruism or reputation-based partner choice^5,6^ (i.e., “I help you now so that I look good and others will choose me in the future”).

These mechanisms rely on reputation (“a characteristic or an attribute that partners ascribe to the actor”^7^), which itself relies on the availability of information about other people’s behaviors. It can be difficult and costly, however, to acquire first-hand reputational information as this requires time and might cause individuals to run the risk of being exploited. Gossip, defined as a sender communicating to a receiver about a target who is absent or unaware of the content^8^, has been argued to have evolved as a less risky and costly alternative to gather reputational information^9^. Gossip implies that knowledge of individuals’ (un)cooperative behavior flows from one individual to another, thus magnifying the available reputational information. As such, gossip enriches the opinions individuals have of others they interact with and provides information about others they never interacted with, creating a wider pool of information for people to draw from when deciding to cooperate or not.

In line with this, a growing body of literature across different disciplines portrays gossip as a means to promote and maintain cooperation^10–13^. Findings show that gossip sustains group cooperation by punishing and isolating free-riders, or by ostracizing group members^10,14–16^. Gossip also allows uncooperative behaviors to be easily identified as it spreads reputational information and enables people to protect themselves against exploitative individuals^15,17,18^. Thus, gossip enables individuals to avoid defective partners and select cooperative ones^12,19^. It has also been proposed that gossip, compared to direct observation, is a less costly yet efficient tool to build reputation and improve social control, defined as the detection and punishment of defectors^12,20^. Hence, an increasing number of studies points towards the beneficial role of gossip, suggesting that it should be endorsed to sustain cooperation. Studies supporting this claim, however, have ignored two important issues.

First, most of the empirical studies compare situations with reputation that was formed through gossip to situations in which no reputation was available, as when participants interact with strangers^10,11,17,19^. This comparison makes it hard to disentangle whether the beneficial role of gossip is due to the spread of gossip or to the introduction of a reputation system. In the real world, gossip generally happens in situations in which people can also, to some extent, observe other people’s behavior, rather than between complete strangers^9,21^. This means that individuals build reputation about others on the basis of both their direct observations (first-hand reputation) and the gossip they receive (second-hand reputation). Comparing the role of first- versus second-hand reputation is essential in disentangling the effect of gossip on cooperation.

To date, only a few studies have addressed this concern, and their results are conflicting. Early studies conducted by Sommerfeld and colleagues investigated how direct observation versus gossip affected cooperation. They found that gossip decreased cooperation compared to first-hand reputation, that is, when individuals based their decisions solely on direct observations^22^. In a second study, however, they found that gossip increased cooperation compared to when direct observation was available^23^.

Overall, their work suggests that while single gossip statements based on incomplete information decrease cooperation^22^, either single or multiple gossip statements based on complete information increase cooperation^23^. In our everyday life, however, as argued by Sommerfeld and colleagues, gossip is very unlikely to reflect complete information. On the contrary, people are likely to encounter multiple gossip statements about others, and these statements are usually based on the limited information that the sender holds on the target, rather than on an accurate history of the target’s behavior. Nevertheless, this situation was not tested by the authors. In a more recent study, Fehr and colleagues showed that gossip increased cooperation compared to first-hand reputation in iterated trust games^12^. However, the gossip and first-hand reputation treatments differed on multiple dimensions in their study, and the authors argued that this comparison should be seen as exploratory.

Here, we present an agent-based model in which agents receive multiple gossip statements about others and base their gossip on the previous encounter they had with the target rather than on the complete history of the target’s behavior. As such, we aim to offer the missing piece of the puzzle that Sommerfeld and colleagues tried to find, providing a more realistic picture of how gossip is spread^22,23^. Thus, our first research question is formulated as follows:

R1: Does gossip foster cooperation compared to first-hand information?

Second, empirical studies have mainly elicited and thus analyzed the role of pro-social gossip (that is, gossip that aims to help the receiver) in cooperation, disregarding the impact of other types of gossip. Research has highlighted multiple reasons for people to engage in gossip^24,25^: to help and protect others, to enhance their own reputation at someone else’s expense, to create bonds with others, to vent emotions, and to enjoy themselves with others. Although these different reasons may give rise to various types of information being shared via gossip, previous studies have tended to focus on gossip that is shared to help receivers, so-called pro-social gossip^17^. Thus, they ignored the role of selfish and exploitative gossip, with the result that knowledge of how gossip affects cooperation is based on only one of its multiple facets^26–28^.

Gossip shared for reasons other than prosocial ones, for example, could vary with respect to the veracity of information sent. That is, if the aim is to help the gossip receiver, gossip might contain truthful information about whether the gossip target behaved cooperatively or not in a previous interaction. However, if the goal is to harm the gossip target (for example to increase the gossip sender’s relative position in the group), gossip could contain lies about the target’s behavior, stating that the target behaved uncooperatively whereas, in reality, the target cooperated.

Fonseca and Peters explored the impact of the accuracy of gossip on cooperation in a repeated two-player trust game in which information about a player’s behavior was transmitted by their previous partner to a new partner either via gossip or via accurate factual summaries. They found that exogenously introduced inaccurate gossip (i.e., gossip that was distorted by the experimenter such that it reflected inaccurate information) decreased cooperation compared to when participants received an accurate factual summary of their partners’ previous behavior. When inaccuracy emerged endogenously (i.e., when participants lied in their gossip messages), however, such lies did not hinder but rather fostered cooperation^26^.

Analysis of the content of the gossip showed, however, that most individuals lied for prosocial reasons (that is, they exaggerated the gossip to punish defectors or to help the gossip receiver), while only a minority of individuals misrepresented information to harm the gossip receiver^27^. Similarly, Fehr and Sutter found in their study that the majority of the gossip contained truthful information (75$\%$), and only 7$\%$ reported untruthful information, while the remaining 18$\%$ of the messages were unclear. It is important to point out, however, that the participants in these studies had no good reasons to send inaccurate gossip. There were, in other words, no incentives to lie.

To examine to what extent situational incentives can affect the accuracy of gossip, Peters and Fonseca^27^ manipulated whether participants were competing with each other or not and analyzed how this affected the frequency of spontaneous lies shared through gossip in an iterated trust game. Whereas they again found that participants mainly lied to help the gossip receivers (by exaggerating gossip targets’ cooperative or defecting behaviors) rather than to harm them (by exchanging information likely to decrease the receivers’ outcome), they also found that gossipers lied twice as often under competition^27^. That people can engage in gossip for selfish reasons also resonates with studies investigating gossip in the context of sexual competition which found that individuals are more likely to gossip to their own advantage when competing for mates^29^. Moreover, studies sampling gossip statements from the general population show a diversity of motives for people to engage in gossip^21^.

Overall, previous studies have lacked in eliciting and incentivizing, and therefore investigating gossip shared for motives other than pro-social ones, that is, those encouraging cooperation and helping receivers to avoid defectors. Different gossip behaviors, however, might be driven by different motives, and they might have different effects on group cooperation^30^. The question how the multiplicity of motives to gossip impacts cooperation has as yet remained unanswered. Thus, our second research question is the following:

R2. Does the influence of gossip on group cooperation depend on the motives that drive it?

As the above two issues impede a full understanding of whether gossip is an effective means to foster cooperation, we developed an agent-based model to investigate how the different motives for gossip shape their impact on cooperation, while directly comparing the effect of first-hand reputation (direct observation) to second-hand reputation (gossip).

We adopted a computational model to systematically investigate the impact of factors such as first- vs second-hand reputation, gossip motives, and other factors on cooperation in a controlled environment in which potential confounding factors are ruled out. While this could in theory also be done in a lab experiment, analyzing the impact of every factor separately would require an enormous sample size. A computational model allowed us to test the effects for large population sizes exceeding 50 agents, which would be infeasible in a lab study, and for long periods of time exceeding 400 interactions, which is also infeasible in a lab study.

**Figure 1:**
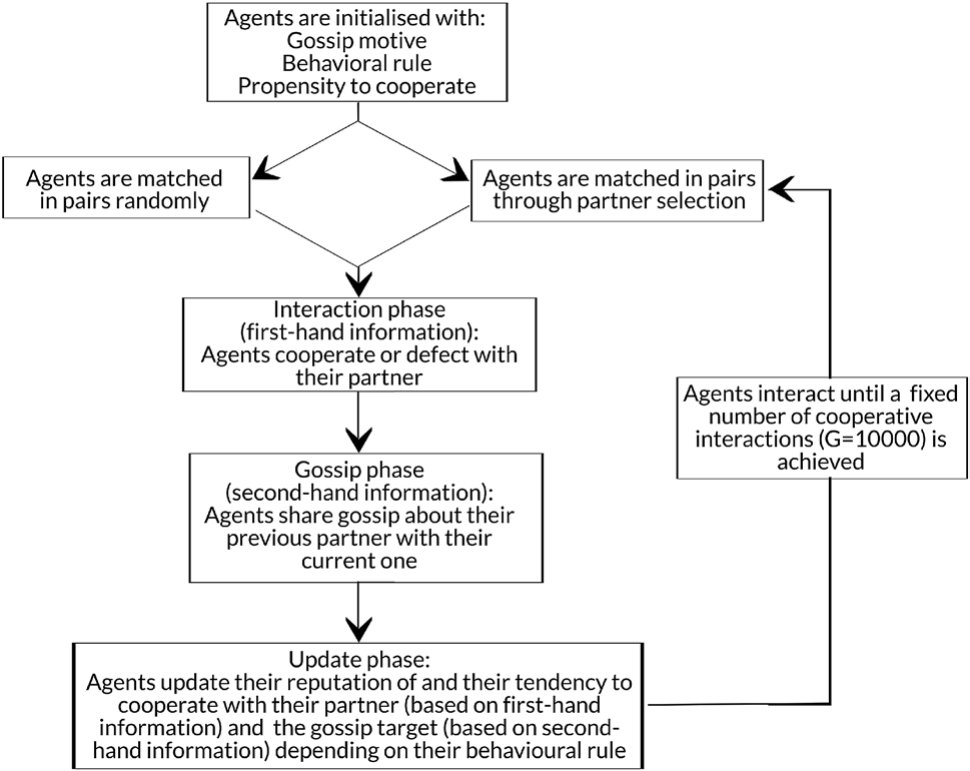
Flowchart of the model dynamics. During initialization, agents are assigned a gossip motive and a behavioral rule that remains constant throughout the entire simulation (see SI, section S1.2 and S1.5). Agents are also initialized with a baseline propensity to cooperate drawn from a normal distribution with variance 5 and mean 50 (see “Methods” section). Agents are either matched with random partners throughout the entire simulation or they select their partner based on their reputation (see “Methods” section and SI, section S1.3). When partner selection is modelled, agents are matched randomly for the first 50 rounds to allow reputational information to be gathered.

**Figure 2:**
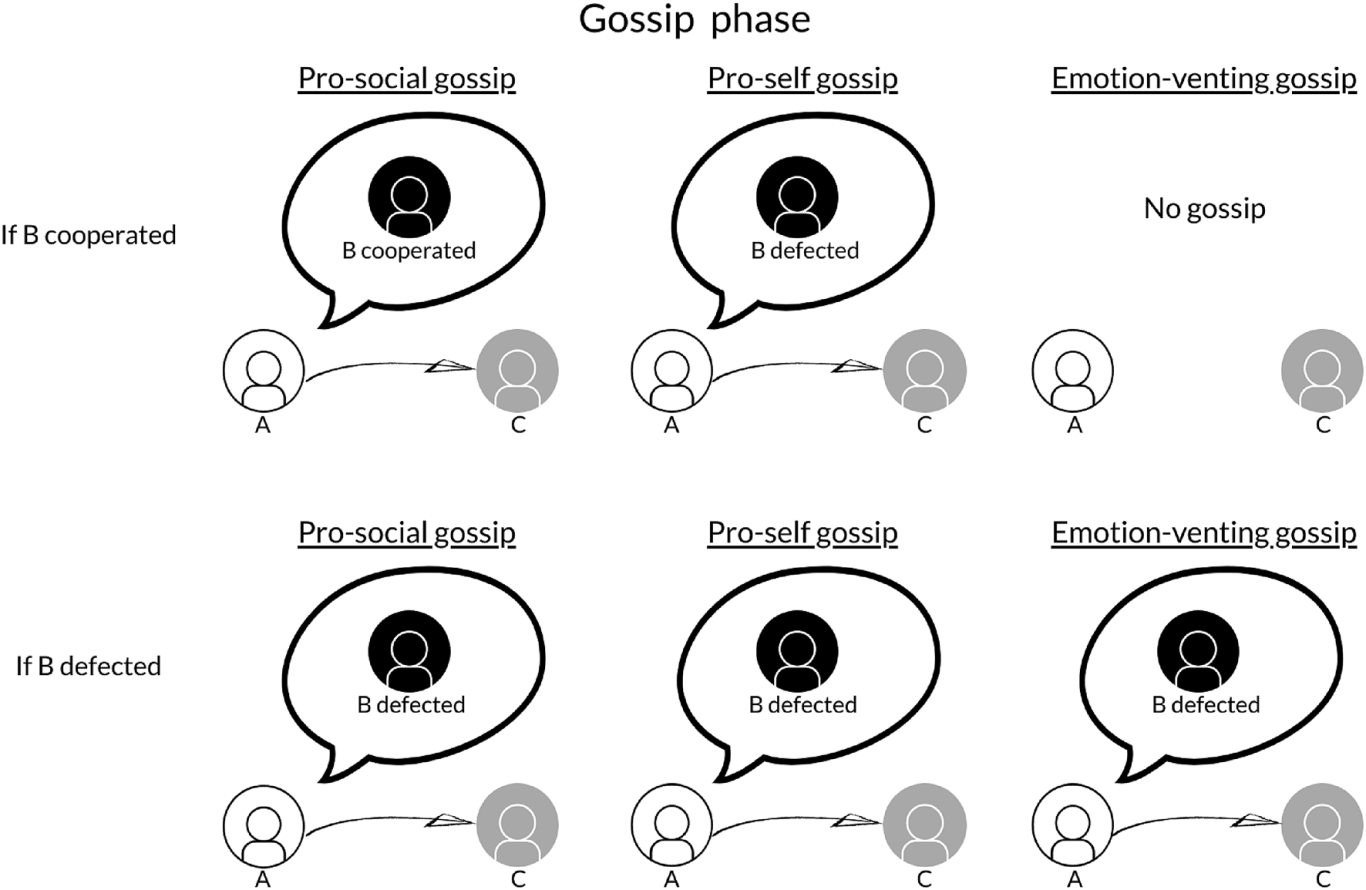
Gossip motives studied in the model. In the Figure, A gossips about B (its previous interaction partner) to C. The content of the gossip depends on A’s gossip motive and B’s behavior in the previous round.

In the model, a population of agents interact in pairs, and each time they interact they have the option to cooperate or defect (agents are initialized with a cooperation frequency drawn from a normal distribution ${\mathcal {N}}(50, 5)$). After making this decision, agents gossip about their previous interaction partner to their current one (see Fig. 1 for an overview of the model flow). The content of the gossip depends on the reason behind it (see Fig. 2). Following the interaction and the gossip phase, agents update their reputation of others and their behavior towards them based both on their direct experiences (first-hand reputation) and on the gossip they received (second-hand reputation). More details about the model and its operationalisation can be found in the “Methods” section and in the SI, section S1.

Our model presents a simplified illustration of the interaction dynamics among humans: when receiving gossip, agents do not evaluate its reliability or its truthfulness based on the gossiper’s reputation; and agents do not change their gossip behavior based on their partner’s reputation. Future models should take these dynamics into consideration to better reflect real-life interactions.

The information that agents share depends on the gossip motive. Possible reasons to gossip have been discussed, and recent classifications distinguish as many as five different motives^24,31^. A first reason to engage in gossip is to maintain the social order and protect one’s group against norm violators (“group protection” motive). That is, people might provide each other with honest, truthful information about the (un)cooperative behavior of their group members because they want them to seek out cooperative others to interact with and to protect them against uncooperative behaviors^12,17^. A second reason might be to shed a negative light on the gossip target’s reputation (“negative influence” motive). People may share information depicting the gossip target as uncooperative to decrease the likelihood of the target being chosen by others as a partner in future interactions, and to increase their own likelihood of being selected as partners, thus creating a competitive advantage^32,33^. A third reason to engage in gossip is to vent one’s emotions (“emotion venting” motive). People may use gossip to share emotionally evocative experiences, and sharing negative events is commonly used to alleviate the negative effects of the past experience^24^. A fourth reason is to create or strengthen relationships (“social enjoyment” motive). By gossiping, people distract themselves from their daily routine and find an alternative way to enjoy spending their time with others, which, it has been argued, cements social bonds between them^9^. Lastly, a fifth reason to gossip is to gather and validate information about others (“information gathering” motive). Individuals may use gossip to “map” those around them, by gathering information about them and validating whether their beliefs about a person are shared by others^32,34^.

Here, we propose that the reasons to engage in gossip are likely to affect gossip content (see Fig. 2 for a representation of these motives as implemented in the model, and SI, section S1.5). Agents motivated to protect group members or to gather information should send truthful information about (un)cooperative interactions to their group members. Hence, we modelled “pro-social” gossipers as agents that always report truthfully what happened in their previous interaction, thus protecting their current partner from exploitative behaviors (if the previous partner defected) and encouraging and sustaining cooperative interactions (if the previous partner cooperated). Agents motivated by the desire to increase their own reputation or relative standing in the group, in contrast, should only report that previous partners behaved uncooperatively. Therefore, we modelled “pro-self” gossipers as agents that always report defective behavior of their previous partner, decreasing their reputations in the eye of the receiver. As negative events are weighted more than positive ones, moreover, agents gossiping because they need to vent their emotions should particularly share negative information (i.e., information about uncooperative interactions). Hence, we modelled “emotion-venting” gossipers as agents that share information about previous partners’ uncooperative behavior to release the tension deriving from a previous negative experience. As positive events do not evoke strong negative feelings that need to be vented, however, they do not share any information if the previous partner cooperated. The social enjoyment motive does not elicit any clear behavioral pattern: People may share both positive and negative, truthful and false information about the person they gossip about, without any strategic reason. As we focus in the model on the motives that have the clearest behavioral implications, we did not include the social enjoyment motive.

Moreover, we examined the impact of gossip motives under different conditions. Specifically, we varied: 1) the way in which agents are matched with their partners, and 2) the way in which agents react to the reputational information.

With regard to how agents chose their partners, we modelled two scenarios (see SI, section S1.3): (I) agents were randomly matched in each round, mimicking a real-life situation in which individuals are paired up by a third party to complete a task, such as for example could be the case for individuals working in an organization and paired into work dyads by a supervisor; and (II) agents chose their partner in each round based on the reputational information they have about their group members. This scenario mirrors a real-life situation in which individuals use the reputational information to select cooperators and avoid defectors.

A large branch of the literature has examined how cooperation evolves depending on how individuals update their behaviors based on the information received^35,36^. In the extensive literature on games and strategies, the most frequent behavioral rules (apart from those that are purely cooperative/defective) whereby agents use the information gathered to form a reputation and act upon it^37^ share two core features: forgiveness and leniency^38,39^. Hence, we modelled two behavioral rules (see “Methods” and SI, section S1.2): (a) the conditional cooperation rule, in which agents are more likely to cooperate if their partner previously cooperated and vice-versa; and (b) the lenient rule, in which agents are more likely to cooperate with cooperators and disregard their opponents’ defections, as long as the number of defective actions remains under a fixed threshold.

Taken together, our results suggest that gossip has mostly a negative effect on cooperation when compared to first-hand information. Challenging the widespread conception in the academic literature that gossip boosts cooperation, we found that pro-self and emotion-venting gossip always depleted cooperation, whereas the positive role of pro-social gossip depended on the agents’ initial propensity to cooperate. Moreover, the impact of gossip on cooperation was moderated by (1) how partners were selected (random matching or partner selection), and (2) how agents reacted to reputational information (conditional cooperation or lenient rule).

## Results

### Gossip motives and random matching

**Figure 3:**
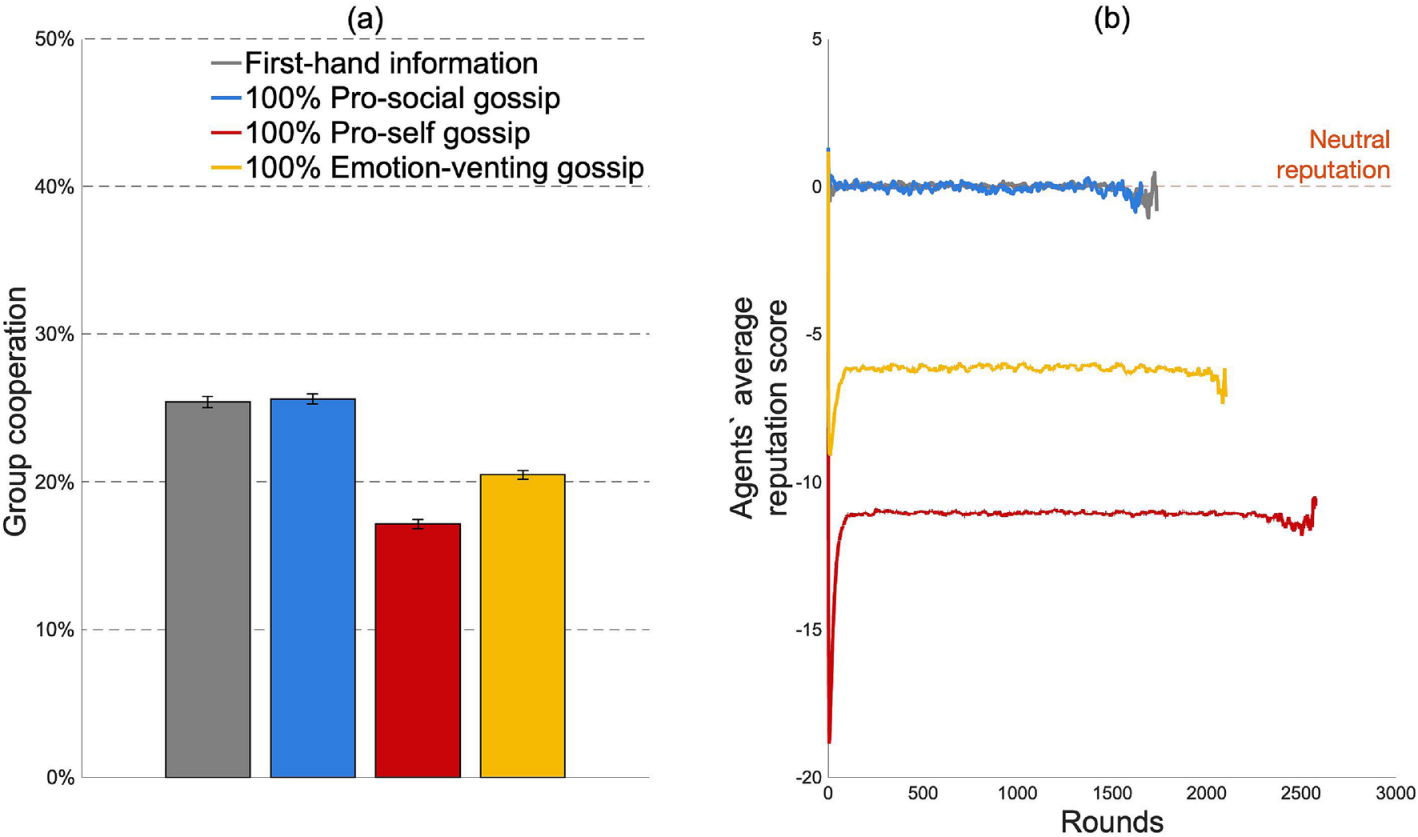
Results for conditionally cooperating agents when they were randomly matched. (**a**) Average group cooperation for first-hand information and gossip motives, (**b**) Agents’ average reputation score of the other group members.

We compared the effect of cooperation when agents built their reputation of others based on (1) first-hand information, and (2) first- and second-hand information, where second-hand information was acquired through gossip. We first present the results when agents were matched randomly and adopted a conditionally cooperating behavioral rule. As shown in Fig. 3a, despite the growing body of literature that depicts gossip as a means to promote and maintain cooperation, gossip did not enhance cooperation compared to first-hand information. Thus, to answer our first research question, first-hand information sustains higher or similar levels of group cooperation compared to gossip (pro-social vs first-hand information: two-sample t-test, ttest2 in MATLAB, t(98) = 0.42, *p* = 0.67; pro-self vs first-hand information: t(98) = − 53.44, *p* < 0.001; emotion-venting vs first-hand information: t(98) = − 27.54, *p* < 0.001; see Fig. 3a). When we compared the impact of gossip motives (our second research question), pro-social motives sustained higher group cooperation levels than emotion-venting and pro-self motives (see Fig. 3a).

Figure 3b sheds light on how these findings came about. When pro-self and emotion-venting gossip was spread, agents received more negative information about the other group members. This flow of negative information decreased the agents’ average reputation, which in turns decreased the agents’ propensity to cooperate (Fig. 3b). Therefore, we observed a lower level of group cooperation when pro-self and emotion-venting gossip was shared than when either pro-social or no gossip was shared. When agents gossiped pro-socially, they received truthful and complete information about their group members (no information was omitted as in the case of emotion-venting gossip). Similarly, when they did not gossip, they updated their reputation based on what they experienced, which is truthful by definition. Thus, in both the pro-social gossip and the first-hand information treatments, agents acquired truthful information about their group members. The only difference is that when pro-social gossip was shared, the amount of information was doubled as information was acquired both through direct interaction and through gossip. As agents were initialized with a 50$\%$ chance to cooperate, they received positive information (i.e., information that previous partners cooperated) and negative information (i.e., information that previous partners defected) about others with the same frequency. Positive and negative information, hence, cancelled each other out, causing group members to have a neutral reputation of one another (Fig. 3b). As the propensity to cooperate with an agent is a linear function of the agent’s reputation, we observed similar cooperation levels in groups with pro-social gossip and first-hand information. If this line of reasoning holds, a different initial propensity to cooperate should lead to differences between the pro-social gossip and first-hand information treatment with regards to agents’ reputation and group cooperation.

In the following, we initialized agents either as high cooperators (their initial chance to cooperate was drawn from a normal distribution with mean of 70$\%$ and variance of 5) or as low cooperators (mean of 30$\%$ and variance of 5), and we varied the percentage of high and low cooperators in each group (see “Methods” section for more details).

**Figure 4:**
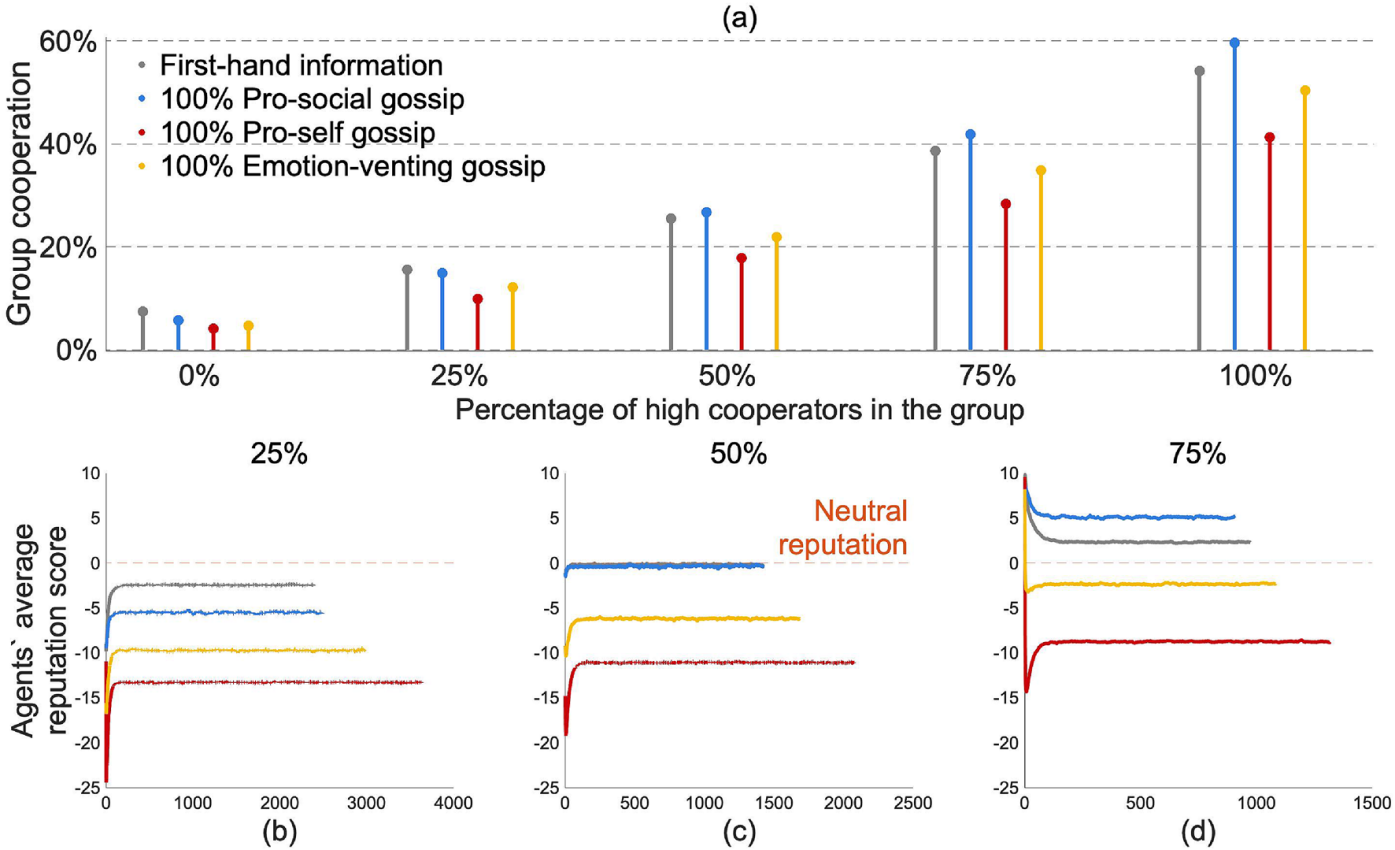
Results for conditionally cooperating agents when they were randomly matched and the group varied with respect to the agents’ initial propensity to cooperate. (**a**) Average group cooperation for different percentages of high cooperators in the group. High cooperators have an initial chance to cooperate around 70$\%$ (normal distribution ${\mathcal {N}}(70, 5)$); low cooperators have an initial chance to cooperate around 30$\%$ (normal distribution ${\mathcal {N}}(30, 5)$). Agents’ average reputation of the rest of the group when agents were randomly matched, and the group was composed of (**b**) 25$\%$ high cooperators, (**c**) 50$\%$ high cooperators, and (**d**) 75$\%$ high cooperators.

Pro-social gossip sustained higher levels of cooperation than both other gossip motives and first-hand information, but only when there were at least 50$\%$ of high cooperators in the group (Fig. 4a). Thus, if agents were more likely to defect than to cooperate (percentage of high cooperators < 50$\%$) the content of the information transmitted through pro-social gossip was mainly negative, thus decreasing the agents’ reputation and their propensity to cooperate faster than when they only received first-hand information (Fig. 4b). If groups were equally split between high and low cooperators, positive and negative information cancelled out, presenting similar results as those presented above (Figs. 3b, 4c). If agents were more likely to cooperate than to defect, however, a larger amount of positive information circulated in the pro-social gossip treatment than in the first-hand information one. More positive information led to higher reputation (Fig. 4d), which in turn led to a higher propensity to cooperate.

Thus, when agents were matched at random, pro-social gossip fostered higher levels of cooperation than first-hand information but only if most agents were more prone to cooperate than to defect (these results also hold for mixed populations in which agents with multiple gossip motives coexist, see SI, section S3). If agents were more likely to defect, on the other hand, they were better off basing their decisions solely on what they experienced first-hand.

### Gossip motives and partner selection

The above results pertain to the situation in which agents were randomly matched to one another. As in many real-life situations people can decide with whom to interact, we examined the influence of gossip on cooperation when partner selection was in place (see “Methods” section and SI for details on partner selection).

**Figure 5:**
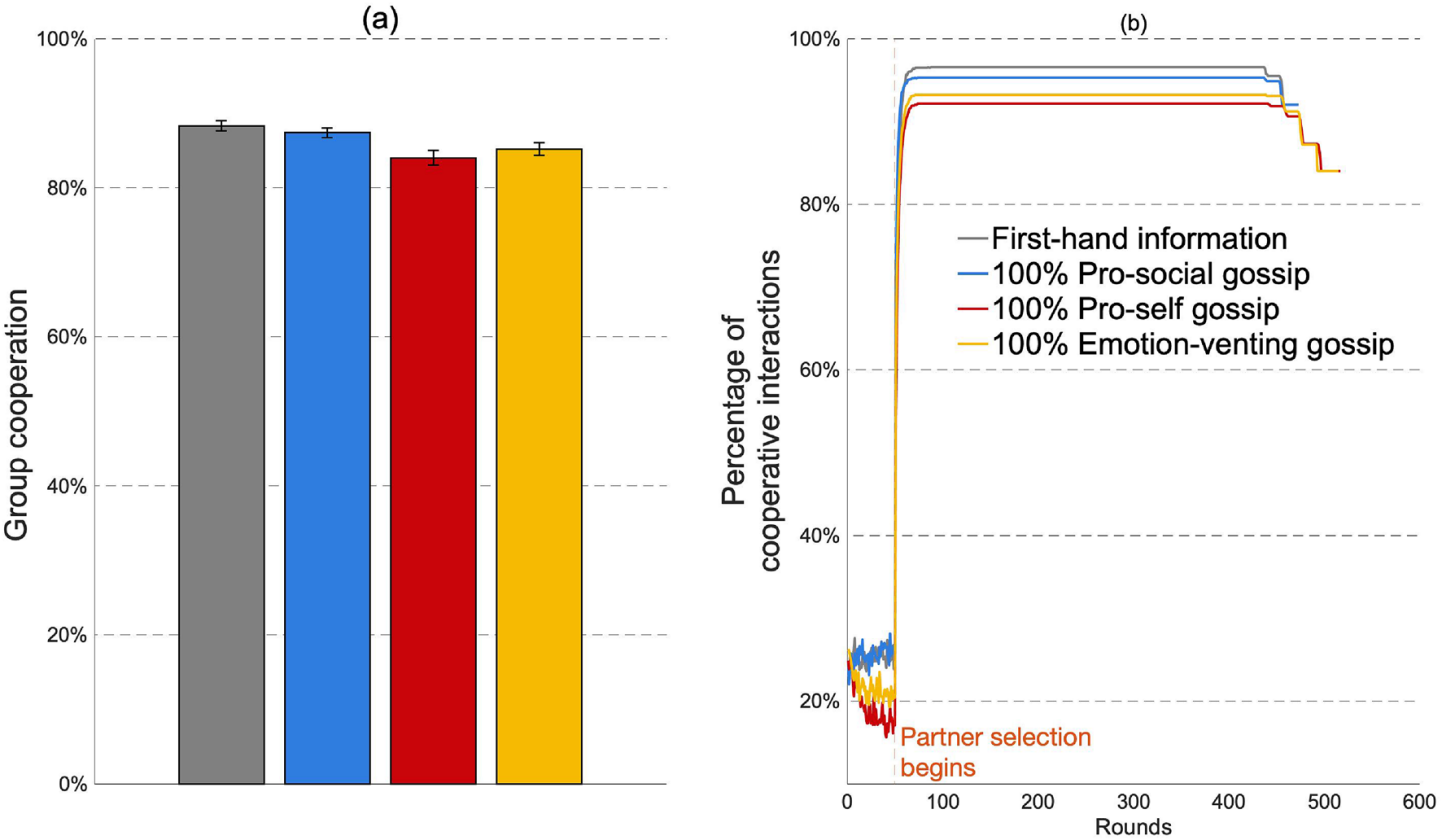
Results for conditionally cooperating agents when they selected their preferred partner. (**a**) Average group cooperation for first-hand information and gossip motives, (**b**) Percentage of cooperative interactions over rounds for first-hand information and gossip motives.

Regardless of the gossip motives examined, partner selection strongly increased group cooperation compared to random matching (from 15–25$\%$ in random matching to 85–90$\%$ in partner selection; see Figs. 3a, 5a). Indeed, when agents selected their partners, they cooperated in most interactions (reaching almost 100$\%$ of cooperation over rounds; see Fig. 5b). When agents could interact with their preferred partners, that is, with those holding a high reputation, their propensity to cooperate with each other increased, reinforcing the positive reputation of their partner, and thus increasing the likelihood that they would select each other in the next interaction (percentage of times agents change their partners: random matching vs partner selection, pro-social gossip: t(98) = 207, *p* < 0.0001; pro-self gossip: t(98) = 154, *p* < 0.0001). This significantly boosted group cooperation while decreasing the differences in cooperation between the gossip motives.

### Lenient behavioral rule

**Figure 6:**
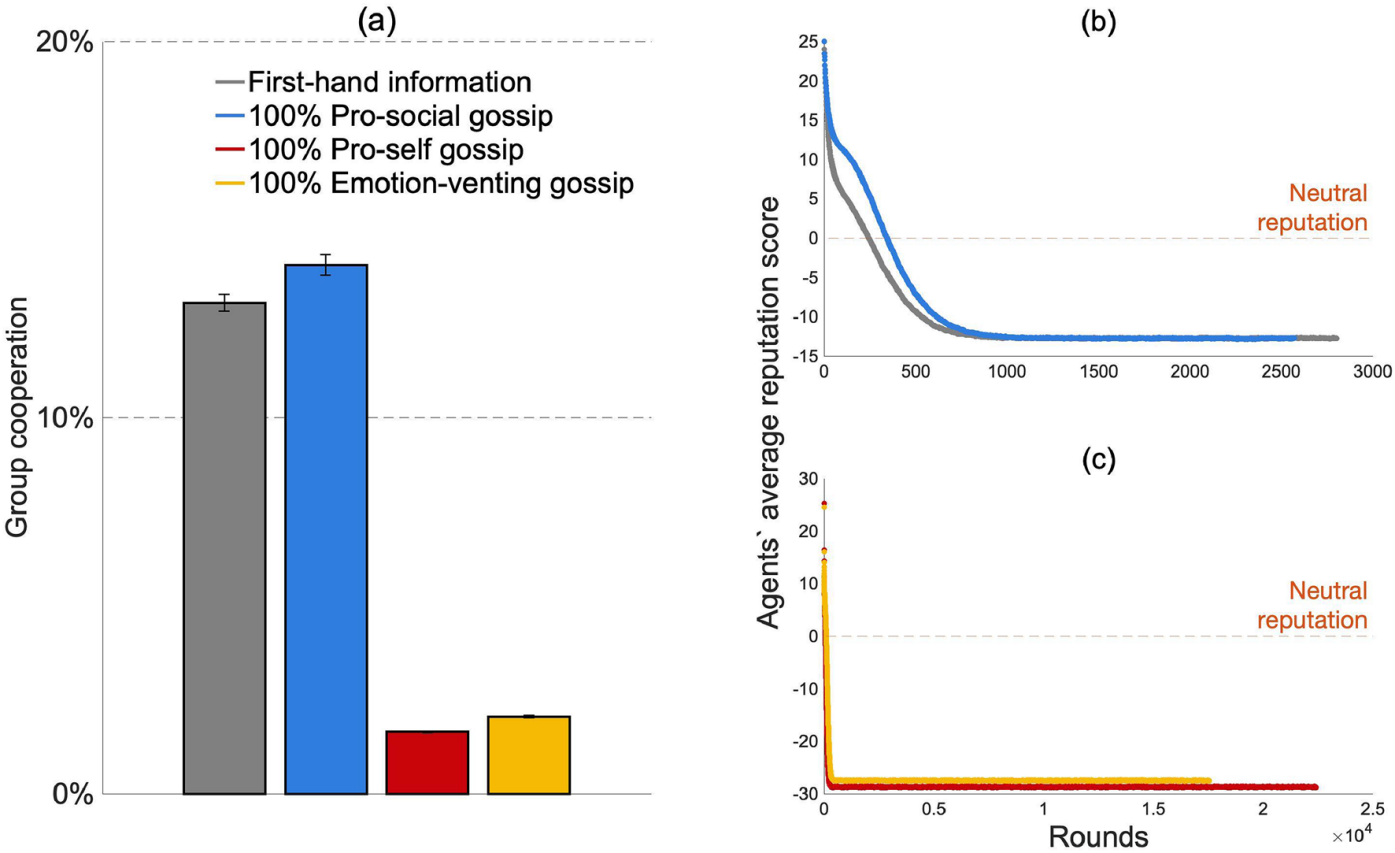
Results for lenient agents when they were randomly matched. (**a**) Average group cooperation for first-hand information and gossip motives. Number of cooperative interactions over rounds for groups (**b**) with first-hand information and groups with 100$\%$ pro-social gossip, and (**c**) with 100$\%$ pro-self and 100$\%$ emotion-venting gossip.

To corroborate our findings, we tested the impact of a different behavioral rule from conditional cooperation: the lenient rule (see “Methods” and SI for a description of the rule and its operationalization). As for conditionally cooperating agents, pro-social gossip motives boosted group cooperation more than either pro-self or emotion-venting ones (see Fig. 6a). However, the effect of gossip motives was considerably stronger for lenient actors than for conditional cooperators (see Figs. 3a, 6a). The low cooperation level observed for pro-self and emotion-venting gossipers under the lenient rule can be explained by the agents’ average reputation over rounds (Fig. 6c). As agents shared a high amount of negative gossip in these treatments, agents reached the threshold of three negative pieces of information (after which agents stop responding to their partner’s cooperative actions and only decrease their probability to cooperate, see “Methods” section and SI for further information) rapidly, drastically decreasing their reputations and their propensity to cooperate. Pro-social gossip motives, on the other hand, increased cooperation, also when compared to first-hand information. Agents in pro-social gossiping groups shared and received more pieces of information than agents in first-hand information groups. While negative information was initially disregarded, positive information enhanced the reputations of their group members and, therefore, their cooperation (as seen in Fig. 6b in the first 500 rounds). Nevertheless, the probability to reach the threshold of three negative pieces of information increased over rounds, and agents would have a negative reputation of most of their team members (as seen in Fig. 6b around 1000 rounds). Pro-social gossip, therefore, provides an initial benefit to group cooperation. When agents selected their partner, results for agents following the lenient rule were similar to those for conditional cooperators: Gossip motives changed group cooperation only slightly, as each group reached a stable level of cooperation (close to 100$\%$, see SI for more details).

Overall, when agents met randomly, our results show the double function of gossip as both a promoter and a deterrent of cooperation in comparison to first-hand information. Our findings show that the role of gossip strictly depends on the motive for gossiping and on the initial propensity of agents to cooperate.

## Discussion

In everyday life, gossip is often portrayed as a negative phenomenon that deters cooperation and is detrimental to the community^40,41^. However, a growing body of literature has highlighted that gossip can be motivated by the desire to warn others and to punish norm-violators^22,23^, thus pointing to a potentially beneficial role of gossip in promoting cooperation^10–13^. This discrepancy between the lay and the scientific portrayal of gossip reveals how complex the phenomenon gossip is: Gossip can serve to enhance one’s reputation at the cost of others and as such it can lead to the spread of false information (which is in line with the lay conceptualization of gossip), but it can also facilitate the identification of norm-violators and their consequent punishment (which is in line with the current scientific conceptualization of gossip).

Previous studies examining the effects of gossip on cooperation, however, had not yet compared how cooperation is affected by different patterns of gossip content driven by different motives to gossip. Additionally, most studies suggesting that gossip increases cooperation have compared gossip to treatments in which no reputation was present^10,11,17,19^, thus impeding a direct assessment of whether it is actually gossip that promotes cooperation, or rather the introduction of a reputation system. In our study, we shed light on the effects of the different gossip motives on cooperation, while also comparing first-hand reputation, gathered through direct interaction, and second-hand reputation, gathered through gossip.

First, our simulation suggests that cooperation increases drastically if agents choose with whom to interact, regardless of which type of gossip circulates in the group. This result corroborates the large body of literature that indicates partner selection as an effective means to promote cooperation^5,6,12,19^.

Secondly, when agents are matched randomly, we found that the effect of gossip on cooperation, compared to agents having access to only first-hand information, is mainly negative, which is in contrast with the widespread notion that gossip promotes cooperation. When agents gossiped negatively about their partners, as they would if they had a pro-self motive, or in other words, if they gossiped to benefit themselves or to vent their emotions, group cooperation strongly decreased compared to first-hand information. When gossip was used to spread both positive and negative information about partners (i.e., the behavioral pattern that fits agents with a pro-social motive), we found that gossip promoted cooperation compared to first-hand information, but only when agents were mostly cooperative.

The beneficial role of gossip in promoting cooperation relies on the extra information that gossip makes available: Gossip increases the flow of information when direct interactions in real-life are difficult or rare. This larger pool of information enables people to identify cooperators and defectors and reciprocate their behavior. While our findings show that gossip indeed increases the amount of available information, they also point out an important aspect: the role of gossip content. When agents lie and only report that others are uncooperative (as in the case of pro-self gossip), the agents’ propensity to cooperate will drop. Even if gossip is truthful (as in the case of pro-social gossip), if most group members defect, most of the available information will be negative, thus decreasing agents’ propensity to cooperate. If most group members cooperate, on the other hand, truthful gossip will spread positive information in the group, which in turn will promote cooperative behaviors among group members, and cooperation will rise. The increase of information through gossip, therefore, does not automatically increase cooperation, as is often postulated: cooperation may increase or decrease depending on the content of the gossip. To summarize, our study leads to two main hypotheses: (1) if people gossip to benefit themselves through lies or if they only share negative information, gossip decreases cooperation compared to first-hand information; (2) if people gossip truthfully, this leads to higher levels of cooperation than first-hand information, but only if the majority of agents are cooperative.

We argue that previous studies have not detected this relation between the content of the information transmitted through gossip and the increase and decrease of cooperation because they hardly ever incentivized manipulative and self-enhancing lies. Indeed, studies that investigated the role of lies in gossip mainly observed the impact of pro-social lies on cooperation, leaving the role of more selfish lies unexplored^15,26,27^. Nevertheless, previous findings showed that people do lie to achieve higher reputational rankings^42^. Our findings, furthermore, point to a side-effect of gossip that most previous empirical studies have failed to consider. Besides supporting the idea that gossip can foster cooperation under some circumstances, we show that, if lying is fruitful for individuals or if group behavior is largely uncooperative, gossip can lead to negative reputational information and may, therefore, decrease cooperation. Our results shed light on both “faces of gossip”: as predicted by lay people, gossip can be detrimental as it facilitates the spread of lies and encourages negative behaviors, and gossip may reinforce positive loops of cooperation at the same time, thus fostering pro-sociality.

Future models could investigate how other reputational rules impact the evolution of cooperation (see for example Ohtuski et al. 2006^35^) as well as how results change if agents critically assess the veracity and the reliability of the gossip received and modify their responses to gossip based on these assessments. For example, agents might take into account the reputation they already have of their partners and either decide not to share information with defectors or not to use the information they receive from defectors. The sustainability of pro-social gossip should also be further examined in an evolutionary model: while we found that agents achieve similar individual gains across gossip motives in mixed populations (see SI, section S3), it is crucial to investigate whether pro-social gossip would lead to evolutionarily stable strategies. Taking these aspects into account would strongly improve the model’s realism by providing a more comprehensive depiction of real-life interactions.

Our results have important implications for how to manage gossip. First, they show that the positive effects of gossip on cooperation may be much more limited than has previously been assumed in empirical studies; a small positive effect of gossip is to be expected only in groups in which group members have a high propensity to cooperate, and only for gossip that is motivated by pro-social motives. These findings show that the effect of stimulating gossip (cf. Wu et al., 2016^11^) in organizations may be less helpful for organizational functioning than previously assumed. Second, our findings corroborate the lay perspective on gossip as a negative phenomenon by showing that the gossip patterns caused by pro-self and emotion-venting motives decrease group cooperation.

An automatic response to these findings might be to call on organizations to attempt to prevent gossip. Earlier research has shown, however, that gossip is very difficult for organizations to prevent gossip as it is often beyond managerial control^43,44^. An alternative way of looking at the present findings points to the importance of educating gossip recipients not to take all gossip at face value and instead to take the gossip sender’s potential motives into account. A recent theoretical model^45^ has argued that gossip recipients are likely to condition their responses to gossip to the motives they attribute to its sender. If this is indeed the case, and recipients are able to distinguish the motives underlying gossip, their response to gossip perceived as prosocial and honest should be different from their response to gossip perceived as pro-self and, therefore, potentially false. This is a potential key for organizations to steer away from the negative effects of pro-self and emotion-venting gossip and towards the positive effects of pro-social gossip. An important avenue for future research, therefore, is to study the impact of recipients’ reactions to gossip.

In conclusion, our findings urge the scientific community to investigate gossip in its entirety by designing experiments in which lies for personal benefits are encouraged and by examining how gossip spread for different reasons impacts group cooperation compared to treatments in which first-hand information is available.

## Methods

### Model

In our model, a population of m agents goes through four stages in each round (see Fig. 1): (1) Matching. Every agent is either randomly matched with another agent or they select their partner according to their reputation score; (2) Interaction. Every agent decides whether to cooperate or defect with their partner; (3) Gossip. Every agent shares a gossip about their most recent interaction partner (different from their current partner) to their current one; (4) Updating. Each agent updates their reputation of and their propensity to cooperate with their partner and the gossip target based on their behavioral rule.

The reputation agents have of each other can be represented in a quadratic $m \times m$ matrix **R**:

1 $$\begin{aligned} \mathbf{R }_{m,m}= \begin{bmatrix} 0 &{} r_{1,2} &{} \cdots &{} r_{1,m}\\ r_{2,1} &{} 0 &{} \cdots &{} r_{2,m}\\ \vdots &{} \vdots &{} \ddots &{} \vdots \\ r_{m,1} &{} r_{m,2} &{} \cdots &{} 0\\ \end{bmatrix}, \end{aligned}$$

where the diagonal represents the reputation each agent has of itself. Each row represents the reputation each agent has of the other group members, while each column represents the reputation that the group holds of one agent.

Agents’ initial propensity to cooperate $c_{i,0}$ is drawn from a normal distribution ${\mathcal {N}}(50,5)$, with the exception of high (${\mathcal {N}}(70,5)$) and low cooperators (${\mathcal {N}}(30,5)$). After each interaction and each gossip received, agents update their propensity to cooperate with their partner and the gossip target.

The way in which agents update their reputation and their propensity to cooperate depends on the behavioural rule adopted. Despite the behavioral rule, the propensity to cooperate (as well as the reputation) is discounted over time (discounting factor = 9/10), meaning that the newer the information is, the more weight it has. More information can be found in the SI, section S1.

### Conditional cooperation rule

When two agents interact, they increase their propensity to cooperate if their partner cooperated and decrease it otherwise. If two agents *i,j* interact at round *t*, agent *i* will update its propensity to cooperate with agent *j* as presented in Eq. (2).

2 $$\begin{aligned} c_{i,j,t+1,obs}&= {\left\{ \begin{array}{ll} +\omega _{coop,obs} &{}\quad \text {if agent j cooperated at time t} \\ -\omega _{coop,obs} &{}\quad \text {if agent j defected at time t} \end{array}\right.} \end{aligned}$$

### Lenient rule

Lenient agents tolerate a fixed number of defections before they react to them (specifically, we implemented a threshold of 3 negative pieces of information). That is, agents increase their cooperation with those that previously cooperated and keep their cooperation constant with those that previously defected. However, if the threshold of 3 defections is achieved, agents stop responding to their partner’s cooperative actions and only decrease their probability to cooperate after each defective piece of information they receive. Therefore, if two agents *i,j* interact at round *t*, agent *i* will update its propensity to cooperate with agent *j* as presented in Eq. (3).

3 $$\begin{aligned} \begin{aligned} c_{i,j,t+1, obs}&= {\left\{ \begin{array}{ll} +\omega _{coop,obs} &{}\quad \text {if agent j cooperated at time t and } def_{i,j} \le 3 \\ 0 &{}\quad \text {if agent j cooperated at time t and } def_{i,j}> 3 \\ 0 &{}\quad \text {if agent j defected at time t and } def_{i,j} \le 3\\ - c_{i,0} - {\tilde{c}}_{i,j,t} - 2500 &{}\quad \text {if agent j defected at time t and } def_{i,j} > 3 \end{array}\right. } \end{aligned} \end{aligned}$$

where ${\tilde{c}}_{i,j,t} = \sum _{k=0}^{t}\left( \frac{9}{10}\right) ^{t+1-k} c_{i,j,k, obs} + \sum _{k=0}^{t}\left( \frac{9}{10}\right) ^{t+1-k} c_{i,j,k, gos}$, and $def_{i,j}$ is the number of defective pieces of information agent *i* has of agent *j*.

### Gossip phase

Similarly, when agent *i* receives gossip about agent *j* about an interaction occurred at time *k*, agent *i* updates its propensity to cooperate as follows in equation (4) for conditional cooperating agents and Eq. (5) for lenient agents.

4 $$\begin{aligned} \begin{aligned} c_{i,j,t+1, gos}&= {\left\{ \begin{array}{ll} +\frac{9}{10}^{(t-k)}*\omega _{coop,gos} &{}\quad \text {if agent j cooperated at time k} \\ -\frac{9}{10}^{(t-k)}*\omega _{coop,gos} &{}\quad \text {if agent j defected at time k} \end{array}\right. } \\ \end{aligned} \end{aligned}$$

5 $$\begin{aligned} \begin{aligned} c_{i,j,t+1, gos}&= {\left\{ \begin{array}{ll} +\frac{9}{10}^{(t-k)}*\omega _{coop,gos} &{}\quad \text {if agent j cooperated at time k and } def_{i,j} \le 3 \\ 0 &{}\quad \text {if agent j cooperated at time k and } def_{i,j}> 3 \\ 0 &{}\quad \text {if agent j defected at time k and } def_{i,j} \le 3\\ 0 &{}\quad \text {if agent j defected at time k and } def_{i,j}> 3 \\ &{}\quad \text {and } c_{i,j,t+1, obs} = - c_{i,0} - {\tilde{c}}_{i,j,t} - 2500\\ - c_{i,0} - {\tilde{c}}_{i,j,t} - c_{i,j,t+1, obs} - 2500 &{}\quad \text {if agent j defected at time k and } def_{i,j} > 3\\ &{}\quad \text {and } c_{i,j,t+1, obs} \ne - c_{i,0} - {\tilde{c}}_{i,j,t} - 2500\\ \end{array}\right. } \end{aligned} \end{aligned}$$

Thus, agent *i*’s cooperative tendency when matched at time *t+1* with agent *j* ($c_{i,j,t+1}$) is a combination of all pieces of information agent *i* holds about agent *j* up to time *t*, discounted over time.

6 $$\begin{aligned} \begin{aligned} c_{i,j,t+1}&= c_{i,0} + {\tilde{c}}_{i,j,t} + c_{i,j,t+1, obs} + c_{i,j,t+1, gos} \\&= c_{i,0} + \sum _{k=0}^{t}\left( \frac{9}{10}\right) ^{t+1-k} c_{i,j,k, obs} + \sum _{k=0}^{t}\left( \frac{9}{10}\right) ^{t+1-k} c_{i,j,k, gos} + c_{i,j,t+1, obs} + c_{i,j,t+1, gos}\\ \end{aligned} \end{aligned}$$

When $c_{i,j,t+1}$ is used to calculate the probability that agent *i* will cooperate with agent *j*, we set $c_{i,j,t+1}$ to range from 0 to 100.

### Partner selection

Agents can either be randomly matched with their partner or they can select with whom to interact. Under partner selection, agents are randomly matched for the first 50 rounds. Once agents have collected information about the other agents in their group, either through direct observation or through gossip, they are matched depending on the reputation they have about other members. In each round, agents are matched so that their partner satisfies their ranking preference (see SI for an example):

- First, agents that have each other as their preferred partner (first position in their respective rankings) are matched.
- Second, agents that have each other in either the first or the second positions in their rankings are matched.
- This procedure is iterated until all agents are matched.

### Sensitivity analysis

To further check the robustness and generalizability of our results, we ran additional simulations manipulating the size of the update ($\omega _{coop, obs}, \omega _{coop,gos} \in \{25,50,75\}$), and the population size ($N \in \{25,50,100,150 \}$). Results are consistent with the main findings reported in the paper (complete results in the SI).

## Supplementary Information

Supplementary Information.

## Data Availability

The data that support the findings of this study are available from the corresponding author upon request.
