# Supplementary material for: Gossip promotes cooperation only when it is pro-socially motivated
Source: Sci Rep. 2022 Mar 21;12:4790. doi: 10.1038/s41598-022-08670-7 (PMC8938477; doi:10.1038/s41598-022-08670-7)
Supplement: Supplementary file 1 — Supplementary Information. [file 41598_2022_8670_MOESM1_ESM.pdf]

Supplementary Information
Gossip Promotes Cooperation Only when Pro-socially Motivated.

Martina Testori[1] Charlotte K. Hemelrijk[2] Bianca Beersma[1]

[1]Department of Organizational Sciences, Vrije Universiteit Amsterdam, De Boelelaan 1105, Amsterdam 1081HV, The Netherlands
[2]GELIEF, University of Groningen, Nijenborgh 7, Groningen 9747AG, The Netherlands
Correspondance to: M. Testori, m.testori@vu.nl

S1 Model

A summary of all the parameters can be found in Table S4.

S1.1 Initialisation

Agents are assigned a gossip motive (either pro-self, pro-social or emotion-venting) and an initial value of cooperation $c_{i,0}$ drawn from a normal distribution $\mathcal{N}(50, 5)$:

$$c_i = c_{i,0} \quad \forall i \in N \tag{1}$$

where $c_{i,0}$ is the initial value drawn from $\mathcal{N}(50, 5)$ (normal distribution with mean 50 and variance 5).

S1.2 Behavioural rules

Agents are assigned a rule which determines how agents use the information they acquire. Based on the work on strategies and repeated games presented by Dal Bó and Fréchette [2] that points out which rules are the most frequently used, we study two rules: Conditional cooperation and lenient rule (see Table S1).

The first rule (conditional cooperation) implies that agents increase their likelihood to repeat what their partner did in the previous interaction. Thus, they increase their cooperation with those that previously cooperated and decrease with those that previously defected. That is, if A cooperates with B, then B will be more likely to cooperate with A in the future. On the contrary, if A defects with B, or B received information about A's defection, then B's likelihood to cooperate with A will decrease. For a more formal description of the rule, see Equation 2.

The second rule (lenient) implies that agents tolerate a fixed number of defections before they react to them. That is, they increase their cooperation with those that previously cooperated and keep their cooperation constant with those that previously defected. However, if the threshold of negative behaviours is surpassed (3 defections), agents stop responding to their partner's cooperative actions and only decrease their probability to cooperate after each defective information they receive. To illustrate, if A defects once with B, B's likelihood to cooperate with A remains unchanged. However, if A defects for more than three times with B, B will stop updating its likelihood to cooperate if A cooperates, and it will decrease its cooperation with A by 2500. This number was selected so that it would take roughly 70 rounds for B to return cooperating with A (if within the 70 rounds B receives negative information about A, B's likelihood to cooperate would decrease again by 2500, delaying the time of cooperation). That is, agent A will forget about the three defections by B and return to cooperating with B according to A's baseline likelihood to cooperate. However, every time A observes that B defects (or receives information about this via gossip, see below), then A's likelihood to cooperate again with B will drastically drop and it

will take again 70 rounds to "forget" this. For a more formal description of the rule, see Equation 3.

Table S1: Rules that agents adopt to process information that is either received through gossip or observed during an interaction.

Rule	Modelling
Conditional cooperation	Agents decrease (increase) their cooperation after their opponent defected (cooperated) or they received information about it through gossip.
Lenient	Agents increase their cooperation after their opponent cooperates or they received information about it through gossip. After the opponent defects 3 times, agents' likelihood to cooperate with the defective opponent drastically drops. Moreover, agents will stop recording any cooperative action (thus they do not increase their likelihood to cooperate after receiving or observing a cooperative action).

S1.3 Matching

All agents are matched in pairs. The mechanism by which they are matched is either random or they select their partner according to their preferences.

S1.3.1 Random matching

Agents are matched with randomly chosen partners throughout the entire simulation.

S1.3.2 Partner selection

Agents are randomly matched for the first r_{random} rounds. Once agents have collected information about the other agents, either through direct observation or through gossip, they are matched depending on the reputation they have about other members. After the first random interactions, each agent has a ranking of the other agents in the group. To illustrate, if agent B has positive information about agent A, then agent B will have a high reputation of agent A. Thus agent A will be at the top of agent B's ranking. Conversely, if agent B has negative information about agent A, then agent B will have a low reputation of agent A. Thus agent A will be at the bottom of agent B's ranking. Partners are selected based on agents' respective rankings (see example below):

- First, agents that are each other's preferred partner (first position in their respective rankings) are matched.

- Second, agents that have each other in either the first or the second position in their ranking are matched.
- This procedure is iterated until all agents are matched.

In each round, agents are matched so that their partner satisfies their ranking preference.

For example, let us suppose that Table S2 is the reputation of the agents in a group, and according to that, each agent has a ranking of whom they would like to be matched with (Table S3).

Table S2: Example of the agents' reputation matrix.

	Agent 1	Agent 2	Agent 3	Agent 4	Agent 5	Agent 6
Agent 1	-	4	-1	0	2	-5
Agent 2	2	-	3	1	6	-4
Agent 3	0	-1	-	-7	3	1
Agent 4	1	-1	5	-	-3	2
Agent 5	-1	3	-2	2	-	5
Agent 6	-5	1	3	-2	5	-

Table S3: Example of the ranking each agent has of their preferred partner.

	First	Second	Third	Fourth	Fifth
Agent 1	Agent 2	Agent 5	Agent 4	Agent 3	Agent 6
Agent 2	Agent 5	Agent 3	Agent 1	Agent 4	Agent 6
Agent 3	Agent 5	Agent 6	Agent 1	Agent 2	Agent 4
Agent 4	Agent 3	Agent 6	Agent 1	Agent 2	Agent 5
Agent 5	Agent 6	Agent 2	Agent 4	Agent 1	Agent 3
Agent 6	Agent 5	Agent 3	Agent 2	Agent 4	Agent 1

Therefore, in this example the agents would be matched as follows: Agent 5 & Agent 6; Agent 1 & Agent 4 ; Agent 3 & Agent 2.

S1.4 Interactions

After agents have been matched, they interact following the behavioral rule they have been assigned to at the beginning (conditional cooperation or lenient rule). If both agents cooperate, the interaction is labelled as cooperative, otherwise as non-cooperative.

After the interaction, each agent updates the reputation score of its partner. The behavioral rule determines how agents will update their reputation of and their cooperation with their partner. Thus, if agent i interacted at round t with agent j, and agent i is a

conditional cooperator, agent i will update its reputation of, and its cooperation tendency with agent j as presented in Equation 2. If agent i is a lenient actor, then the update will follow Equation 3.

$$c_{i,j,t+1,obs} = \begin{cases} +\omega_{coop,obs} & \text{if agent } j \text{ cooperated at time } t \\ -\omega_{coop,obs} & \text{if agent } j \text{ defected at time } t \end{cases} \tag{2}$$

$$c_{i,j,t+1,obs} = \begin{cases} +\omega_{coop,obs} & \text{if agent } j \text{ cooperated at time } t \text{ and } def_{i,j} \leq 3 \\ 0 & \text{if agent } j \text{ cooperated at time } t \text{ and } def_{i,j} > 3 \\ 0 & \text{if agent } j \text{ defected at time } t \text{ and } def_{i,j} \leq 3 \\ -c_{i,0} - \tilde{c}_{i,j,t} - 2500 & \text{if agent } j \text{ defected at time } t \text{ and } def_{i,j} > 3 \end{cases} \tag{3}$$

where $\tilde{c}_{i,j,t} = \sum_{k=0}^{t} \left(\frac{9}{10}\right)^{t+1-k} c_{i,j,k,obs} + \sum_{k=0}^{t} \left(\frac{9}{10}\right)^{t+1-k} c_{i,j,k,gos}$, $def_{i,j}$ is the number of non cooperative pieces of information agent i has about agent j, and $\omega_{coop,obs}$ is the weight with which agents update their cooperative strategy after observing agent j's behavior (default value = 50). By setting the likelihood to cooperate to -2500, agent i takes roughly 70 rounds (with no additional information about that agent) to "forget" the defection of agent j and return to their baseline level of cooperation towards agent j. However, whenever they receive any additional negative information about agent j, then the time to "forget" and to return to its baseline likelihood to cooperate will increase.

The reputation and the tendency to cooperate with another agent are updated in the same way, thus the only difference lies in their initial values. While the initial value of cooperation is drawn from a normal distribution, agents initial reputation score of other members is set to zero. The reputation (and the cooperation tendency) is updated in such a way that more recent events have more impact.

After each interaction individuals update their current personal satisfaction, based on the outcome of the interaction. This measure captures how satisfied (or not) each agent is with its interaction partner; if agents are matched with cooperative partners, they will be more satisfied than when matched with uncooperative partners. Specifically, the personal satisfaction increases by 1 if the opponent cooperated and decreases by 1 if it defected.

$$w_{i,t} = w_{i,t-1} + \begin{cases} +1, & \text{if the opponent cooperated at time } t \\ -1, & \text{if the opponent defected at time } t \end{cases} \tag{4}$$

S1.5 Gossip

Once an interaction has been completed, agents gossip to their current partner about the previous (different) agent they interacted with. The content of the gossip depends on the

gossip motive the agent has been initiated with. The gossip is always perceived as true by the receiver and agents update both their reputation of and their cooperative tendency with the gossip's target following the same rules presented in Equation 5 and 6 depending on their behavioural rule (conditional cooperation and lenient rule respectively).

$$c_{i,j,t+1,gos} = \begin{cases} +\omega_{coop,gos} & \text{if agent } j \text{ cooperated at time } k \\ -\omega_{coop,gos} & \text{if agent } j \text{ defected at time } k \end{cases} \tag{5}$$

$$c_{i,j,t+1,gos} = \begin{cases} +\frac{9}{10}^{(t-k)} * \omega_{coop,gos} & \text{if agent } j \text{ cooperated at time } k \text{ and } def_{i,j} \leq 3 \\ 0 & \text{if agent } j \text{ cooperated at time } k \text{ and } def_{i,j} > 3 \\ 0 & \text{if agent } j \text{ defected at time } k \text{ and } def_{i,j} \leq 3 \\ 0 & \text{if agent } j \text{ defected at time } k \text{ and } def_{i,j} > 3 \\ & \text{and } c_{i,j,t+1,obs} = -c_{i,0} - \tilde{c}_{i,j,t} - 2500 \\ -c_{i,0} - \tilde{c}_{i,j,t} - c_{i,j,t+1,obs} - 2500 & \text{if agent } j \text{ defected at time } k \text{ and } def_{i,j} > 3 \\ & \text{and } c_{i,j,t+1,obs} \neq -c_{i,0} - \tilde{c}_{i,j,t} - 2500 \end{cases} \tag{6}$$

where k is the round at which the interaction on which the gossip is based happened, and $\omega_{coop,gos}$ is the weight with which agents update their cooperative strategy after receiving information about agent j's behavior (default value = 50).

Thus, agent i's cooperative tendency when matched at time $t+1$ with agent j ($c_{i,j,t+1}$) is a combination of all pieces of information agent i holds about agent j up to time t, discounted over time.

$$\begin{aligned} c_{i,j,t+1} &= c_{i,0} + \tilde{c}_{i,j,t} + c_{i,j,t+1,obs} + c_{i,j,t+1,gos} \\ &= c_{i,0} + \sum_{k=0}^{t} \left(\frac{9}{10}\right)^{t+1-k} c_{i,j,k,obs} + \sum_{k=0}^{t} \left(\frac{9}{10}\right)^{t+1-k} c_{i,j,k,gos} + c_{i,j,t+1,obs} + c_{i,j,t+1,gos} \end{aligned} \tag{7}$$

When $c_{i,j,t+1}$ is used to calculate the probability that agent i will cooperate with agent j, we set $c_{i,j,t+1}$ to range from 0 to 100.

S2 Parameters

Table S4 reports the parameters used in the model and experiments. 'Experimental' parameters are varied in the experiments and 'fixed' parameters are kept constant.

Table S4: Model's parameters.

**Parameter description**	Symbol	Value/Range
Experimental parameters		
Mean of the initial level of cooperation (drawn from a normal distribution with $\sigma^2 = 5$)	μ	$\{30, 50, 70\}$
Weight of the update of the propensity to cooperate following direct interactions	$\omega_{coop,obs}$	$\{0, 25, 50, 75\}$
Weight of the update of the propensity to cooperate following gossip statements	$\omega_{coop,gos}$	$\{0, 25, 50, 75\}$
Percentage of pro-social gossipers	P_{soc}	$[0\%, 100\%]$
Percentage of pro-self gossipers	P_{self}	$[0\%, 100\%]$
Percentage of emotion-venting gossipers	P_{ev}	$[0\%, 100\%]$
Fixed parameters		
Group size	N	50
Group goal (number of cooperative interactions)	G	10000
Number of model replicas	I	50
Number of interactions in which agents are randomly matched	r_{random}	N

S3 Mixed populations

In the main manuscript, results showed populations composed of either 100% pro-social gossipers, 100% pro-self gossipers or 100% emotion-venting gossipers. Hereafter, we show the main results when mixing two gossip motives within the same population. Our main findings are supported also when considering multiple gossip motives within a population: First-hand information promotes higher levels of cooperation than any other gossip treatment, when agents have a 50% chance to cooperate with their partners.

S3.1 Conditional cooperators

First, we show the result when agents behave as conditional cooperators. As Figure S1 shows, first-hand information outperforms all gossip treatments. In other words, regardless of the kind of gossip that agents share, if agents only react to what they experience first-hand, cooperation is higher than when they also listen to gossip (panel a).

Figure S1(b) shows that when agents receive information through gossip, the average reputation of the rest of the group is lower, thus explaining the lower level of cooperation overall.

Within the gossip treatments, when pro-social gossip is present, groups perform better than when pro-self and emotion-venting gossipers are combined within one population.

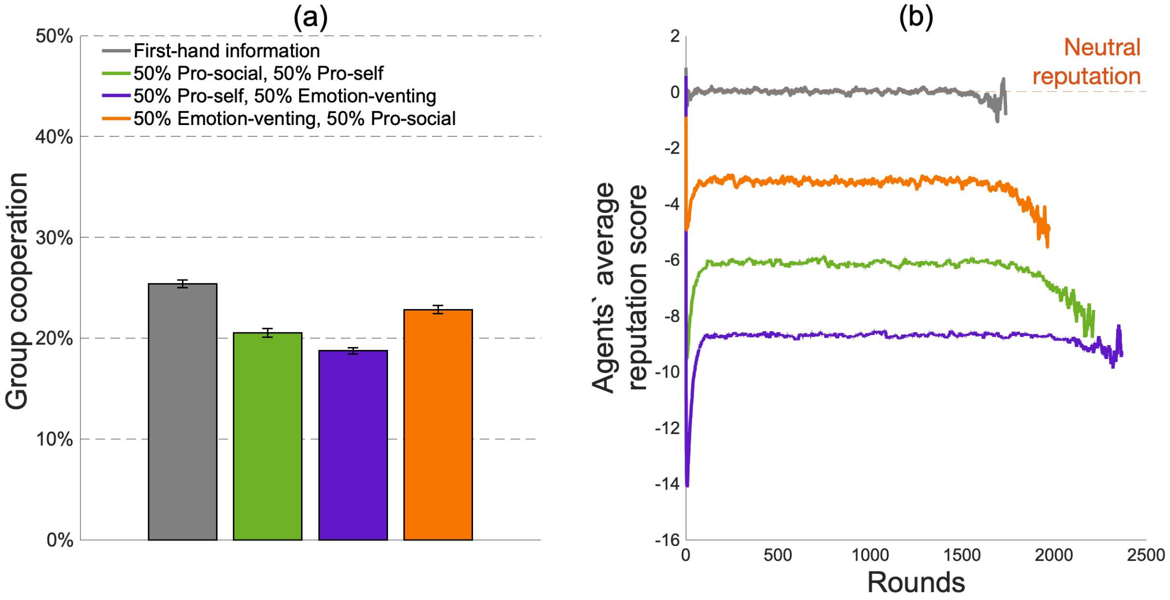

Figure S1: Results for conditionally cooperating agents when they were randomly matched and mixed gossip motives are present within a population. **a**. Average group cooperation for first-hand information and gossip treatments, **b**. Agents' average reputation score of the other group members.

The dynamics presented when considering multiple gossip motives at once are similar to those reported in the main text (Figure 3 main text) when a single gossip motive is present in the population. Since agents receive double the pieces of information (in the gossip treatment compared to the first-hand information only) and they have a 50% chance to cooperate, agents are more likely to decrease their cooperation with other agents when receiving gossip and therefore the group cooperation decreases.

When partner selection is introduced, similar to the results in the main text (Figure 5 in the main text), group cooperation does not depend on the gossip agents receive (see Figure S2).

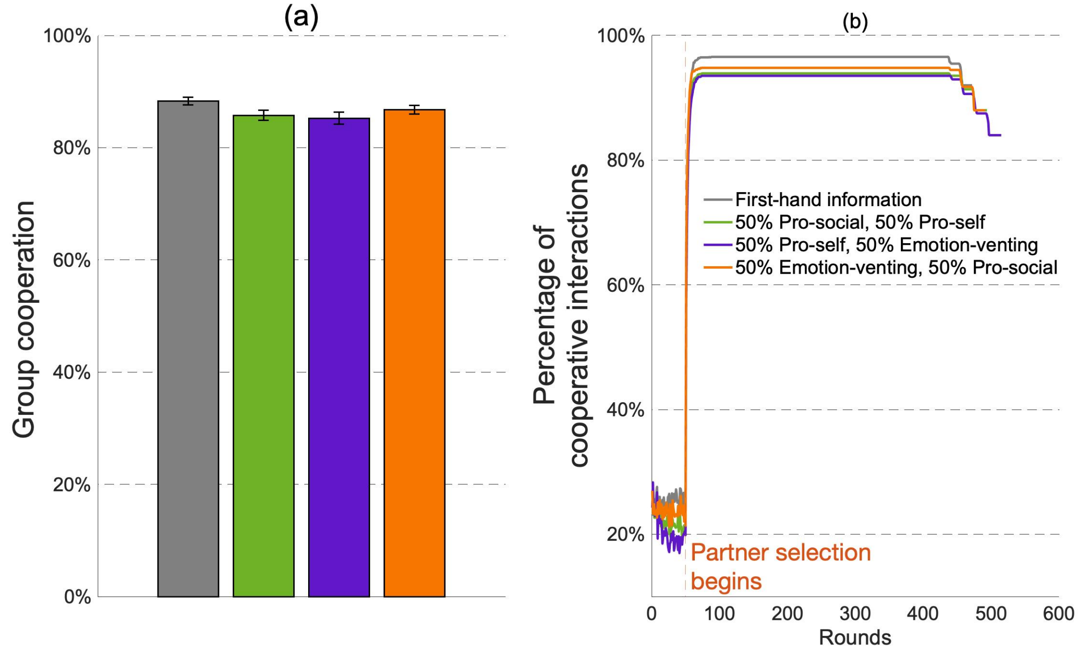

Figure S2: Results for conditionally cooperating agents when they selected their preferred partner and mixed gossip motives are present within a population. **a.** Average group cooperation for first-hand information and gossip treatments, **b.** Number of cooperative interactions over rounds for first-hand information and gossip treatments.

In the random matching scenario, we also explored whether pro-social gossip could be advantageous not only for the group but also for the agents themselves. We did so by comparing the relative individual gain of agents. Based on the payoff structure of the Prisoner's Dilemma[1] (temptation>reward>punishment>sucker), we calculated the individual's gain such that, for each interaction, agents gained: 4 points when they defected and their partner cooperated (temptation), 3 points when they cooperated and their partner cooperated (reward), 2 points when they defected and their partner defected (punishment), and 1 point when they cooperated and their partner defected (sucker). The points were assigned in accordance with the payoff structure of the Prisoner's Dilemma.

We then computed, for each gossip motive, the average agent's gain in each of the conditions above. Results showed that, when agents were matched randomly, agents obtained similar overall scores (50% pro-social, 50% pro-self: $t(98)$=-0.017, p=0.98; 50% emotion-venting, 50%pro-self: $t(98)$=0.001, p=0.99; 50% pro-social, 50% emotion-venting: $t(98)$=0.033, p=0.97). Results for lenient agents also show no differences across agents (50% pro-social, 50% pro-self: $t(98)$=0.038, p=0.97; 50% emotion-venting, 50% pro-self: $t(98)$=-0.001, p=0.99; 50% pro-social, 50% emotion-venting: $t(98)$=0.062, p=;0.95).

This simple measure of individual gain shows that, even though pro-social gossip does

not lead to higher individual gains, it does not lead to lower gains either. Nevertheless, pro-social gossip does entail a benefit for collective outcomes as shown in our analyses that compare groups with pro-social gossipers to groups with either pro-self or emotion-venting gossipers (see the results section in the main manuscript and Figures 3,4). Thus, if we assume that the individual gain is linked to collective gain, then individuals would indeed be better off gossiping pro-socially[3,4].

S3.2 Lenient agents

Figure S3 shows the dynamics for lenient agents. Our results show similar findings as those reported in the section above for conditionally cooperating agents: first-hand information strongly outperforms all gossip treatments. Regardless of the kind of gossip that agents share, if agents only react to what they experience first-hand, cooperation is higher than when they also listen to gossip (panel (a)).

Figure S3(b) explains the results of panel (a) by showing how, when agents receive gossip, the reputation they have of the rest of the group is considerably lower, thus decreasing the overall group cooperation.

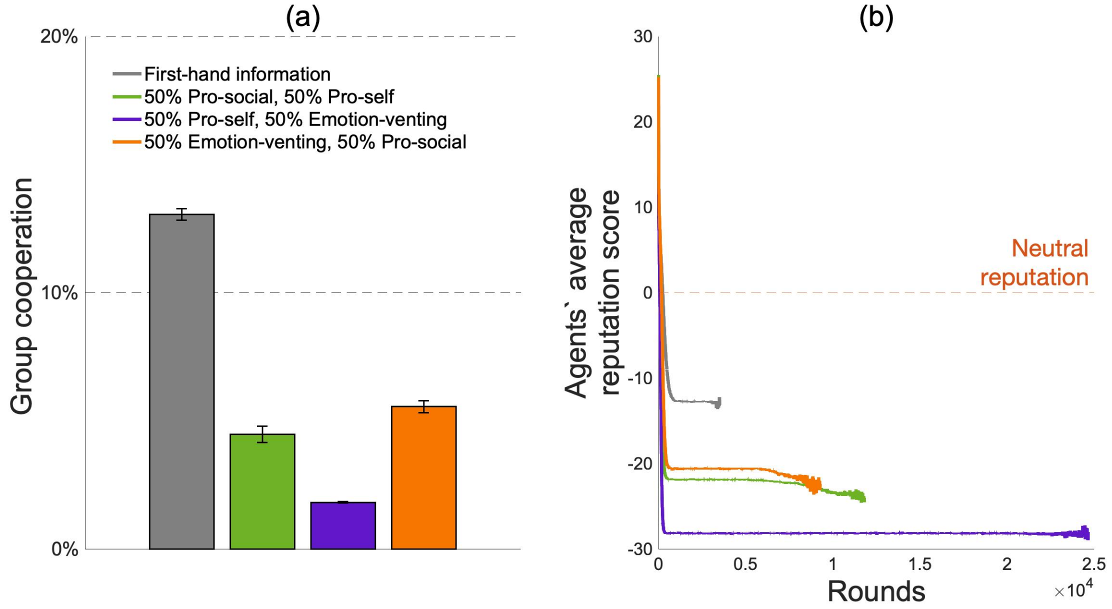

Figure S3: Results for lenient agents when they were randomly matched and mixed gossip motives are present within a population. **a**. Average group cooperation for first-hand information and gossip treatments, **b**. Agents' average reputation score of the other group members.

When partner selection is introduced, similar to the results presented above, group cooperation does not depend on the gossip agents receive (see Figure S4).

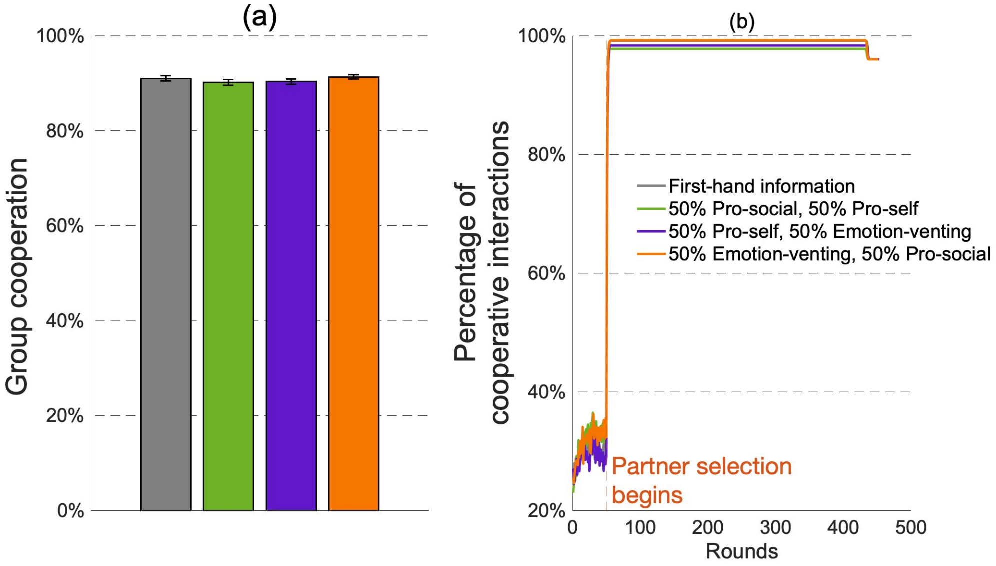

Figure S4: Results for lenient agents when they selected their preferred partner and mixed gossip motives are present within a population. **a.** Average group cooperation for first-hand information and gossip treatments, **b.** Number of cooperative interactions over rounds for first-hand information and gossip treatments.

S4 Sensitivity analysis

S4.1 Agents' initial level of cooperation

The agents' initial propensity to cooperate is drawn from a normal distribution $\mathcal{N}(\mu, \sigma^2)$. While the results in the main paper are reported for $\mu = 50, \sigma^2 = 5$ (meaning that agents have roughly the same chance to cooperate or defect), we here show the results when the initial level of cooperation is either lower or higher than 50%.

Results are consistent with our main findings for random matching (Figure S5). When agents are likely to cooperate ($\mu = 70$), pro-social gossip fosters higher levels of cooperation than the no-gossip condition. On the other hand, when agents are likely to defect ($\mu = 30$), first-hand information leads to higher cooperation levels than any gossip motive. The results are consistent regardless of the behavioural rule adopted (either conditional cooperation or lenient rule).

Figure S5: Group cooperation without (baseline) and with gossip when varying the gossip motives. Agents are randomly matched. Results show the findings depending on the agents' initial level of cooperation drawn from a normal distribution $\mathcal{N}(\mu, 5)$. Panel (a) shows the results for conditional cooperators, and Panel (b) for lenient agents.

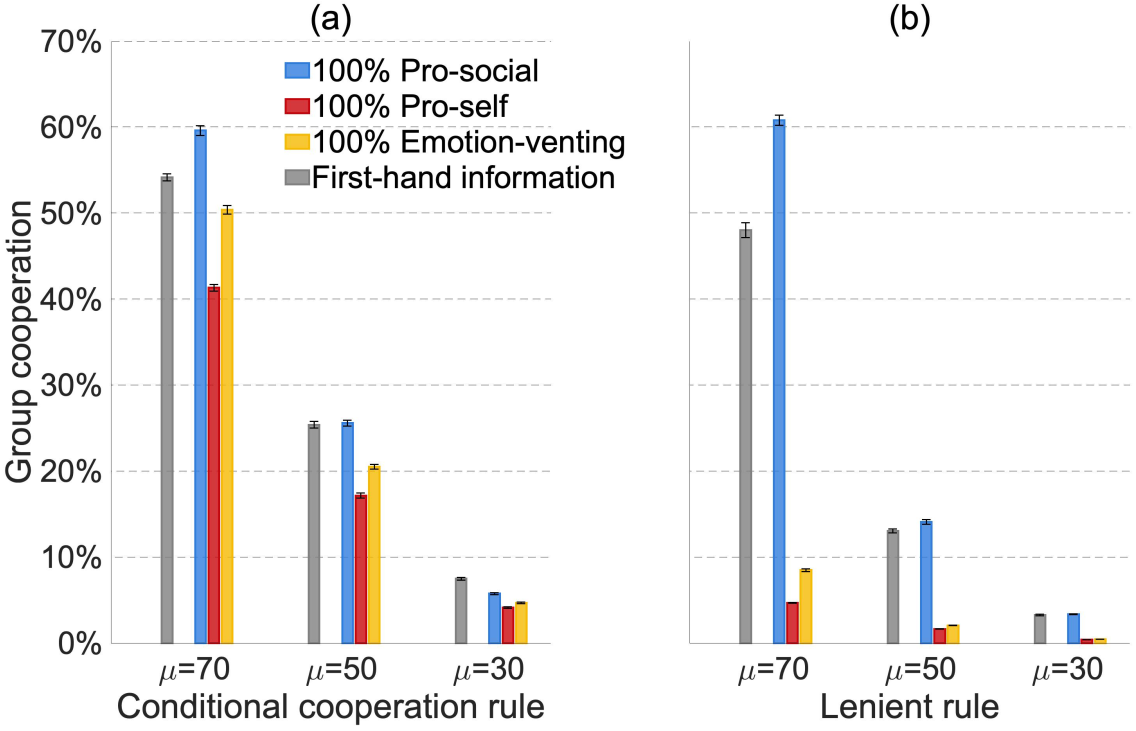

When agents select their partners, results are also consistent with those reported in the main article (Figures S6) as cooperation is higher than for random matching. When the tendency to cooperate of agents is high ($\mu = 70$), pro-social gossip and no gossip result in similar levels of cooperation whether agents act as conditional cooperators (panel (a)) or as lenient actors (panel (b)). When the tendency to cooperate decreases ($\mu = 30$), differences in the behavioural rules emerge: cooperation is higher when agents are lenient actors than when they conditionally cooperate. Agents using a lenient rule are less affected by the amount of negative information circulating in the group since they disregard the first three pieces of negative information they receive. Thus, once they find their preferred partner, lenient agents are not affected by others' actions. Moreover, conditional cooperators do not achieve the high levels of cooperation of lenient agents (see Figure S7).

Figure S6: Group cooperation without (baseline) and with gossip when varying the gossip motives. Agents can choose their partner. Results show the findings depending on the agents' initial level of cooperation drawn from a normal distribution $\mathcal{N}(\mu, 5)$. Panel (a) shows the results for conditional cooperators, and Panel (b) for lenient agents.

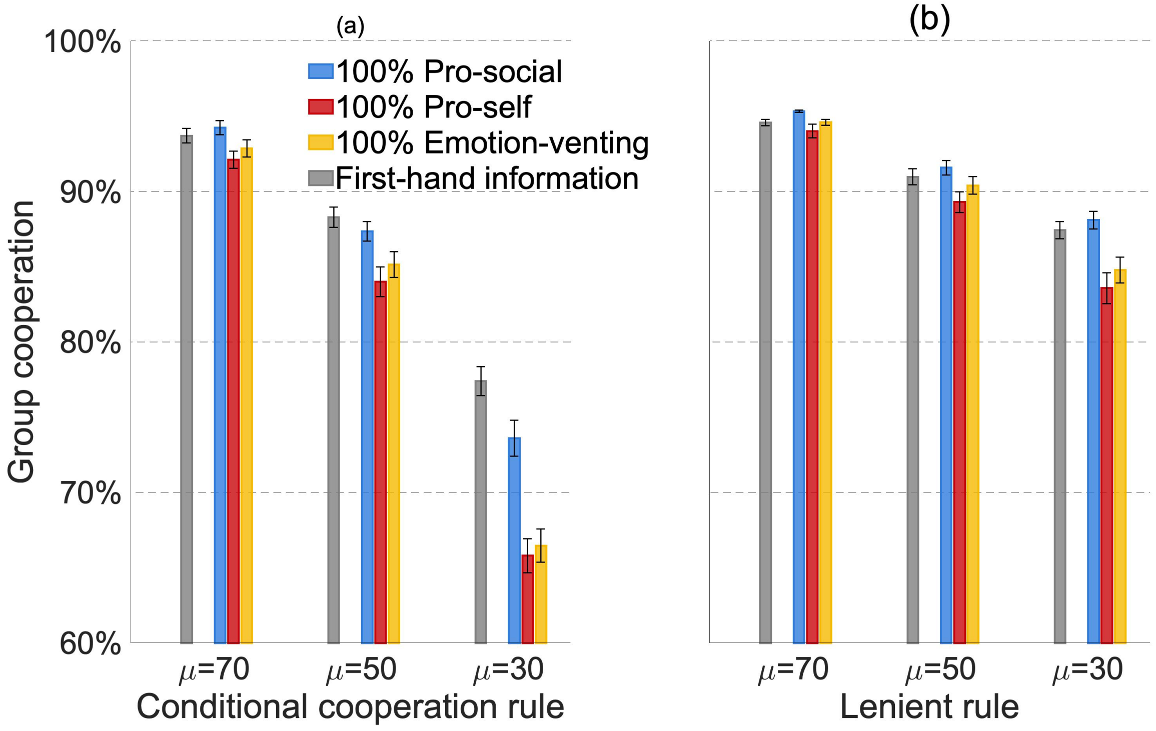

Figure S7: Percentage of cooperative interactions when agents' initial level of cooperation is low (initial level of cooperation drawn from a normal distribution $\mathcal{N}(30, 5)$). Results are presented without (baseline) and with gossip when varying the gossip motives. Agents can choose their partner. Panel (a) shows the results for conditional cooperators, and Panel (b) for lenient agents.

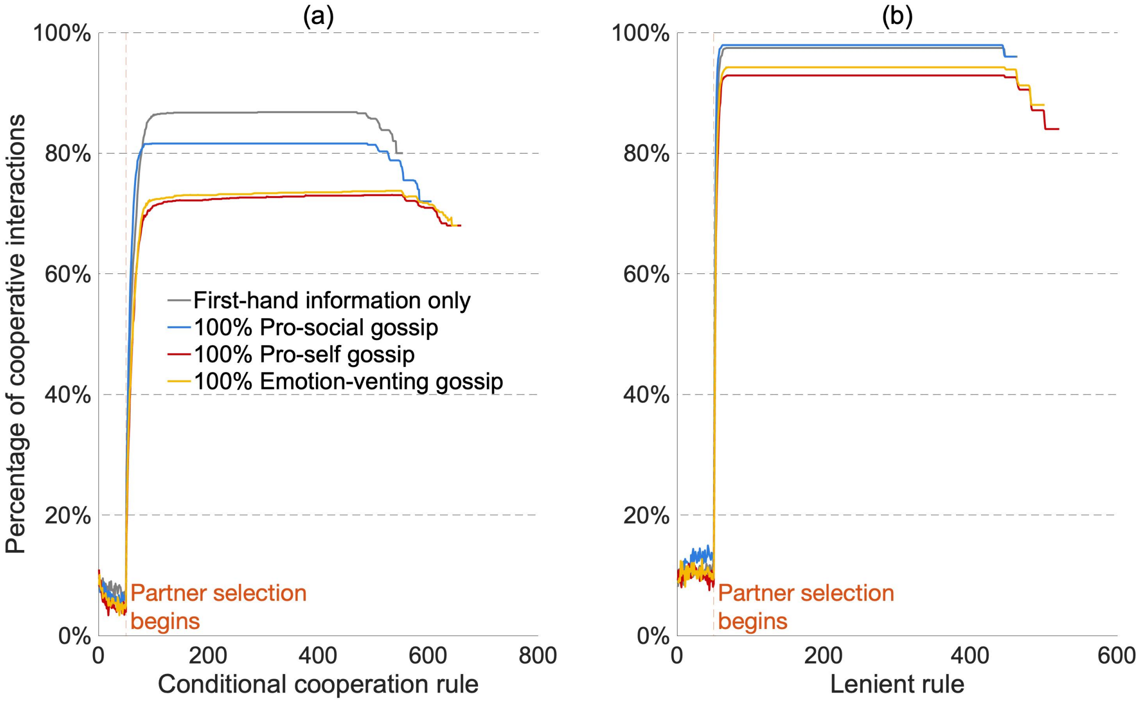

S4.2 Group size

We tested the model for different population sizes. When agents are matched randomly, findings are the same as for conditionally cooperating agents (Figures S8, panel (a)). On the other hand, cooperation increases as population size increases and the lenient rule is adopted (Figures S8, panel (b)). In a larger population, it takes longer to reach the threshold of three defections, thus delaying the decrease of the cooperation, leading to higher group cooperation levels.

Figure S8: Group cooperation without (baseline) and with gossip when varying the gossip motives. Agents are randomly matched. Results show the findings depending on the population size. Panel (a) shows the results for conditional cooperators, and Panel (b) for lenient agents.

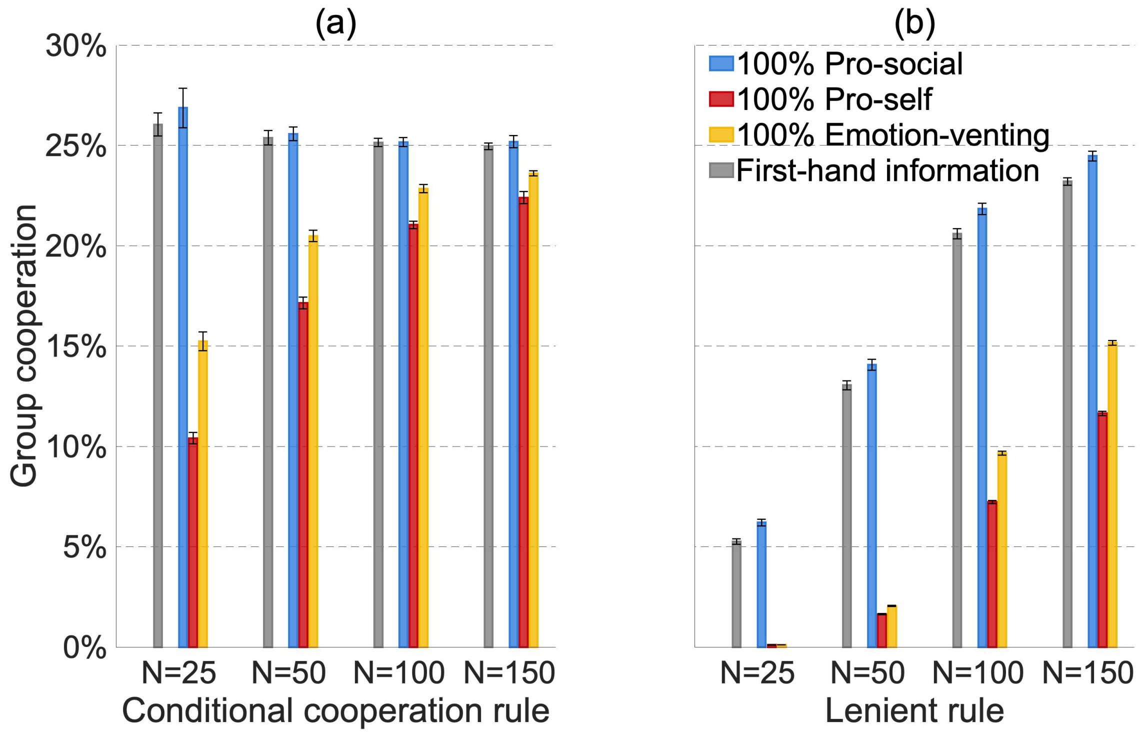

On the other hand, when agents select their partner, cooperation is lower for large populations (Figure S9). The more agents in a group, the more difficult is for agents to find and be matched with their preferred partner, explaining the decrease in cooperation.

Figure S9: Group cooperation without (baseline) and with gossip when varying the gossip motives. Agents can choose their partner. Results show the findings depending on the size of the groups. Panel (a) shows the results for conditional cooperators, and Panel (b) for lenient agents.

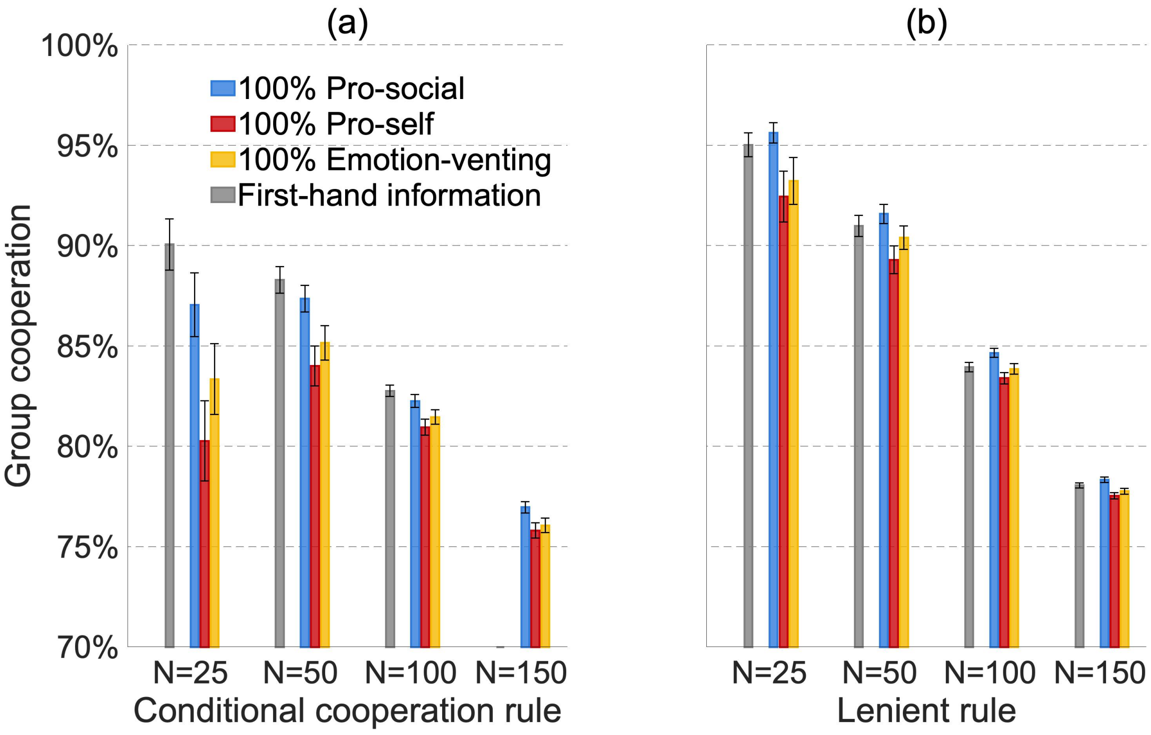

S4.3 Agents' weights to update their cooperation levels and reputation scores

Finally, the size ($\omega_{coop,obs}, \omega_{coop,gos}$) of the increase and decrease of agent's cooperation level and reputation scores do not impact the groups dynamics (see Figure S10, S11). That is, the extent to which agents update their cooperation tendency and their reputation of others following a direct interaction or a gossip does not lead to any difference in the cooperation dynamics analyzed.

Figure S10: Group cooperation without (baseline) and with gossip when varying the gossip motives. Agents are randomly matched. Results show the sensitivity of the findings depending on the weight with which agents update their reputation of and their cooperation towards the other group members. Panel (a) shows the results for conditional cooperators, and Panel (b) for lenient agents.

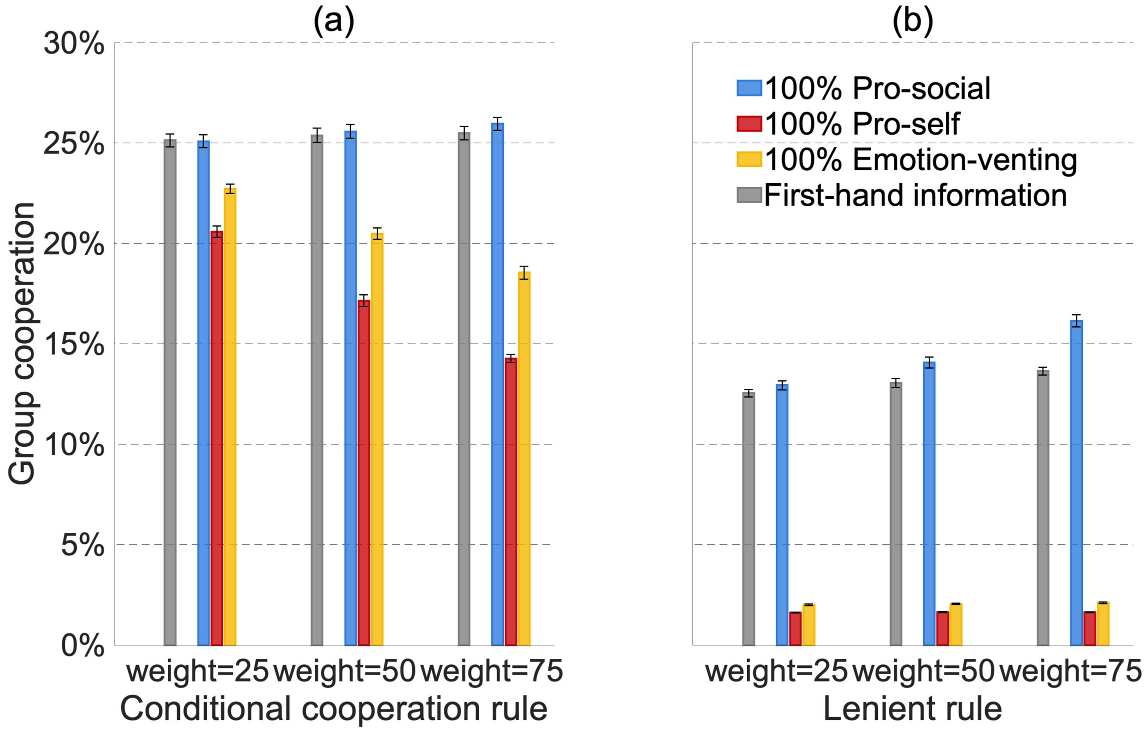

Figure S11: Group cooperation without (baseline) and with gossip when varying the gossip motives. Agents can choose their partner. Results show the sensitivity of the findings depending on the weight with which agents update their reputation of and their cooperation towards the other group members. Panel (a) shows the results for conditional cooperators, and Panel (b) for lenient agents.

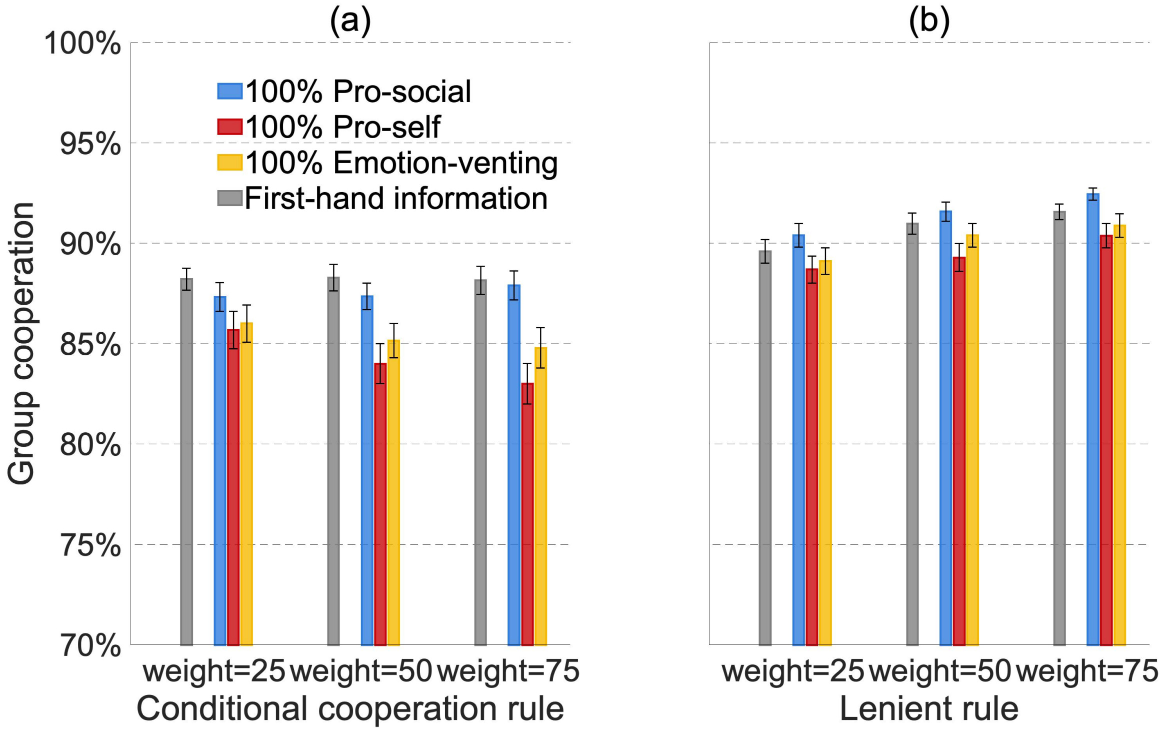

S5 Lenient rule

The following section reports the same plots presented for conditional cooperation in the main paper for the lenient rule.

Figure S12: Results for lenient agents when they are randomly matched.
a. Group cooperation for fist-hand information and gossip conditions,
b. Agents' average reputation score of the other group members.

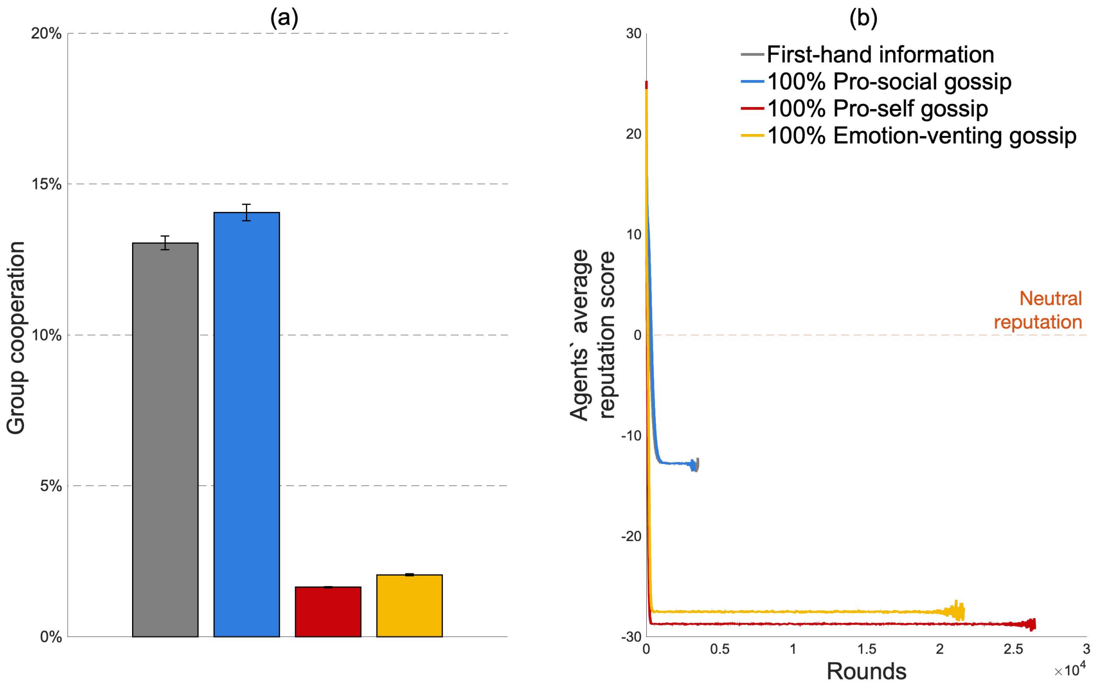

Figure S13: Results for lenient agents when they are randomly matched, and the group varies with respect to the agents' initial propensity to cooperate.
a. Group cooperation for different percentages of high cooperators in the group.
Agents' average reputation of the rest of the group when agents are randomly matched, and the group is composed of **b.** 25% high cooperators, **c.** 50% high cooperators, and **d.** 75% high cooperators.

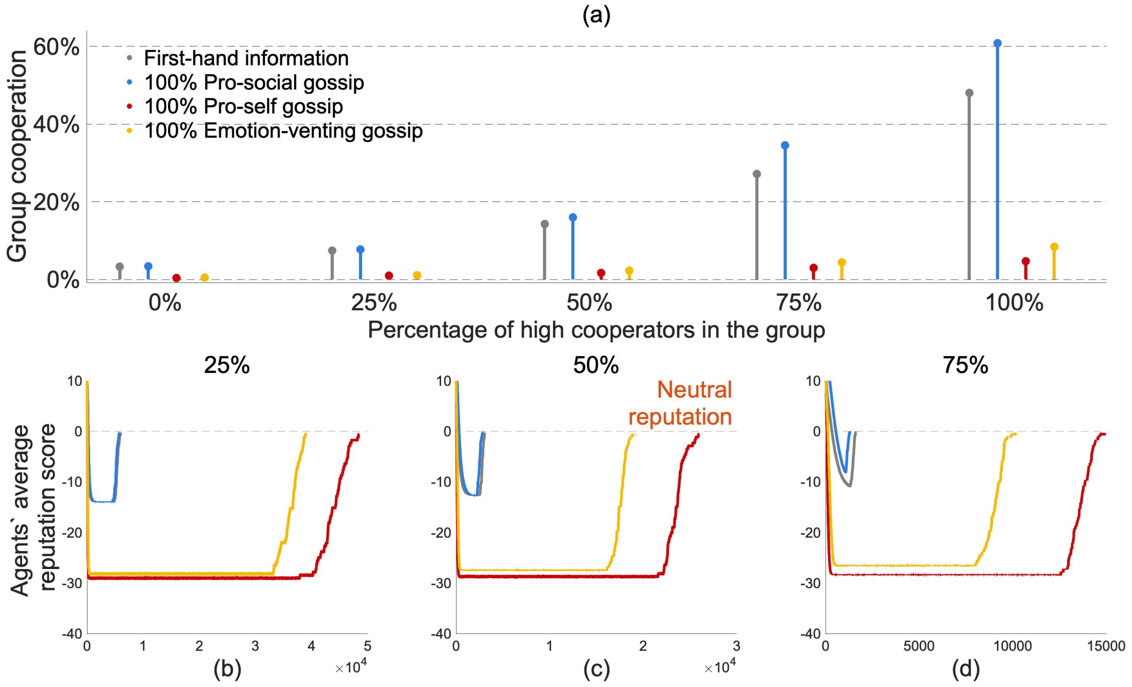

Figure S14: Results for lenient agents when they select their preferred partner.
a. Group cooperation for first-hand information and gossip conditions,
b. Number of cooperative interactions over rounds for first-hand information and gossip conditions.

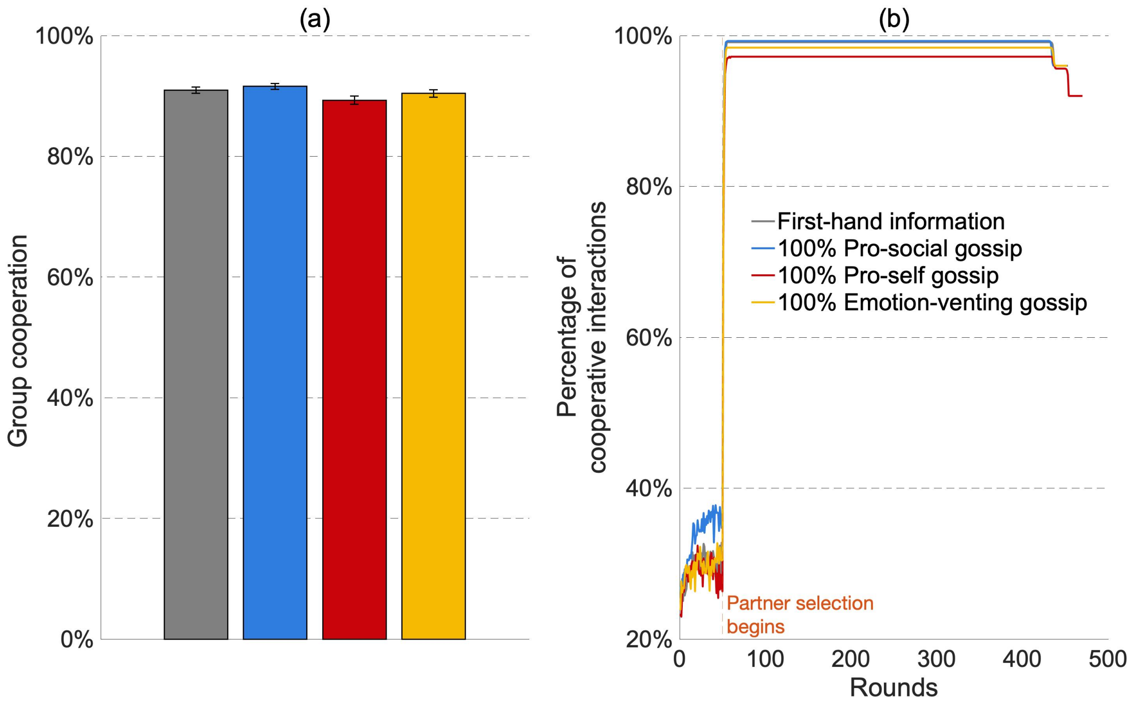

References

[1] R. Axelrod and W. D. Hamilton. The evolution of cooperation. *science*, 211(4489): 1390–1396, 1981.

[2] P. Dal Bó and G. R. Fréchette. The Evolution of Cooperation in Infinetely Repeated Games : Experimental Evidence. *American Economic Review*, 101:411–429, 2011.

[3] S. Okasha. The levels of selection debate: philosophical issues. *Philosophy Compass*, 1 (1):74–85, 2006.

[4] D. S. Wilson. A theory of group selection. *Proceedings of the national academy of sciences*, 72(1):143–146, 1975.
